# Organization of minicircle cassettes and guide RNA genes in *Trypanosoma brucei*

**DOI:** 10.1261/rna.079022.121

**Published:** 2022-07

**Authors:** Sinclair Cooper, Elizabeth S. Wadsworth, Achim Schnaufer, Nicholas J. Savill

**Affiliations:** Institute of Immunology and Infection Research, University of Edinburgh, Edinburgh, Scotland EH9 3FL, United Kingdom

**Keywords:** kinetoplast, minicircle, *Trypanosoma brucei*, guide RNA

## Abstract

Mitochondrial DNA of protists of order *Kinetoplastida* comprises thousands of interlinked circular molecules arranged in a network. There are two types of molecules called minicircles and maxicircles. Minicircles encode guide RNA (gRNA) genes whose transcripts mediate post-transcriptional editing of maxicircle encoded genes. Minicircles are diverse. The human sleeping sickness parasite *Trypanosoma brucei* has one of the most diverse sets of minicircle classes of all studied trypanosomatids with hundreds of different classes, each encoding one to four genes mainly within cassettes framed by 18 bp inverted repeats. A third of cassettes have no identifiable gRNA genes even though their sequence structures are similar to cassettes with identifiable genes. Only recently have almost all minicircle classes for some subspecies and isolates of *T. brucei* been sequenced and annotated with corresponding verification of gRNA expression by small-RNA transcriptome data. These data sets provide a rich resource for understanding the structure of minicircle classes, cassettes and gRNA genes and their transcription. Here, we provide a statistical description of the functionality, expression status, structure and sequence of gRNA genes in a differentiation-competent, laboratory-adapted strain of *T. brucei*. We obtain a clearer definition of what is a gRNA gene. Our analysis supports the idea that many, if not all, cassettes without an identifiable gRNA gene contain decaying remnants of once functional gRNA genes. Finally, we report several new, unexplained discoveries such as the association between cassette position on the minicircle and gene expression and functionality, and the association between gene initiation sequence and anchor position.

## INTRODUCTION

The mitochondrial DNA, called kinetoplast DNA (kDNA), of protists of order *Kinetoplastida* comprises thousands of interlinked, circular DNA molecules, usually in a disk-shaped network. These molecules are of two types: maxicircles and minicircles. Maxicircles, of which there are roughly 20–50 identical copies, are 20–40 kb in length depending on the species (Lukes et al. 2002; Kay et al. 2020). The maxicircle encodes 18 protein components of the mitochondrial respiratory chain, the ATP synthase and the mitoribosome, and two mitoribosomal RNAs (Simpson 1987). Minicircles are diverse with—again depending on the species—dozens to hundreds of different sequence classes, each of which occurs in multiple copies, resulting in about 5000 to 10,000 minicircles per cell (Steinert and van Assel 1980; Cooper et al. 2019). Minicircles encode guide RNA (gRNA) genes of 40–50 bp (Blum et al. 1990; Pollard et al. 1990; Sturm and Simpson 1990; Cooper et al. 2019; see Table 1 for a glossary of terms). Guide RNAs bind to mitochondrial mRNA transcripts using Watson–Crick and GU wobble base-pairing and direct the post-transcriptional U-insertion/deletion editing that is required for 12 of the 18 mRNA transcripts in the kinetoplastid family *Trypanosomatidae* (Benne et al. 1986; Benne 1989; Simpson and Shaw 1989; Stuart 1989; Aphasizhev and Aphasizheva 2014; Read et al. 2016). Each gRNA has a unique 5′ “anchor” region of 6–11 nt length that binds via Watson–Crick base-pairing to preedited and partially edited mRNA transcripts. The central “guiding” region of each gRNA directs U-insertion/deletion editing of the mRNA by virtue of its complementarity to the edited sequence (including G–U wobble base-pairing). Hence, the coding sequence of edited mRNAs is defined by both preedited mRNA and the corresponding gRNAs: The genetic information is split between maxicircle and minicircle sequences. A 3′ oligo(U) tail is post-transcriptionally added to gRNAs and has been suggested to stabilize gRNA–mRNA interaction (Koslowsky et al. 2004). For so-called “pan-edited” mRNA transcripts (which require editing over their entire, or nearly entire, length), subsequent action of numerous gRNAs is required. Here, editing commences with an “initiator” gRNA near the 3′ end of the mRNA and proceeds in a cascade-like fashion where binding of the anchor region of each subsequent gRNA requires complete and correct editing of the editing sites (termed a block) by the previous gRNA. Hence, editing of the mRNA proceeds in an overall 3′ to 5′ direction (Read et al. 2016). Some mRNA species contain only two overlapping editing blocks, but the mechanism is the same. There is one exception to this mechanism. The mRNA transcript that encodes subunit 2 of cytochrome *c* oxidase (COX2) has a single editing block of four inserted U nucleotides. This is guided by a region within the 3′ untranslated region of the mRNA, that is, the gRNA is encoded in *cis* on the maxicircle (Golden and Hajduk 2005). Significant progress has been made in elucidating gRNA biogenesis, identifying the editing apparatus and understanding how editing is controlled (for reviews, see Cruz-Reyes et al. 2018; Aphasizheva et al. 2020). Editing is catalyzed by the so-called “holo-editosome,” comprising over forty proteins found in established macromolecular complexes including the “RNA editing core complex” (RECC), the “RNA-editing catalytic complex” (RESC), the “RNA-editing helicase 2 complex” (REH2C), and several auxiliary factors.

**TABLE 1.**
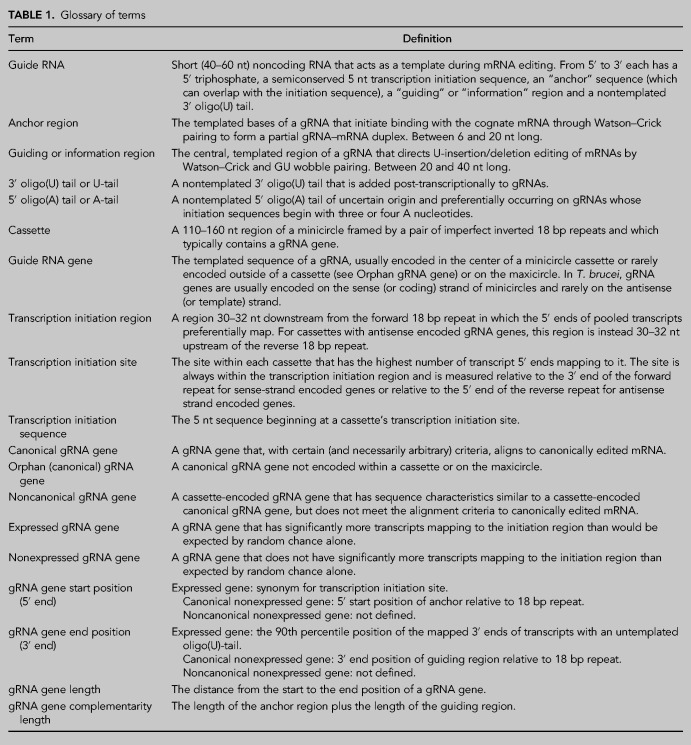
Glossary of terms

**FIGURE 1.**
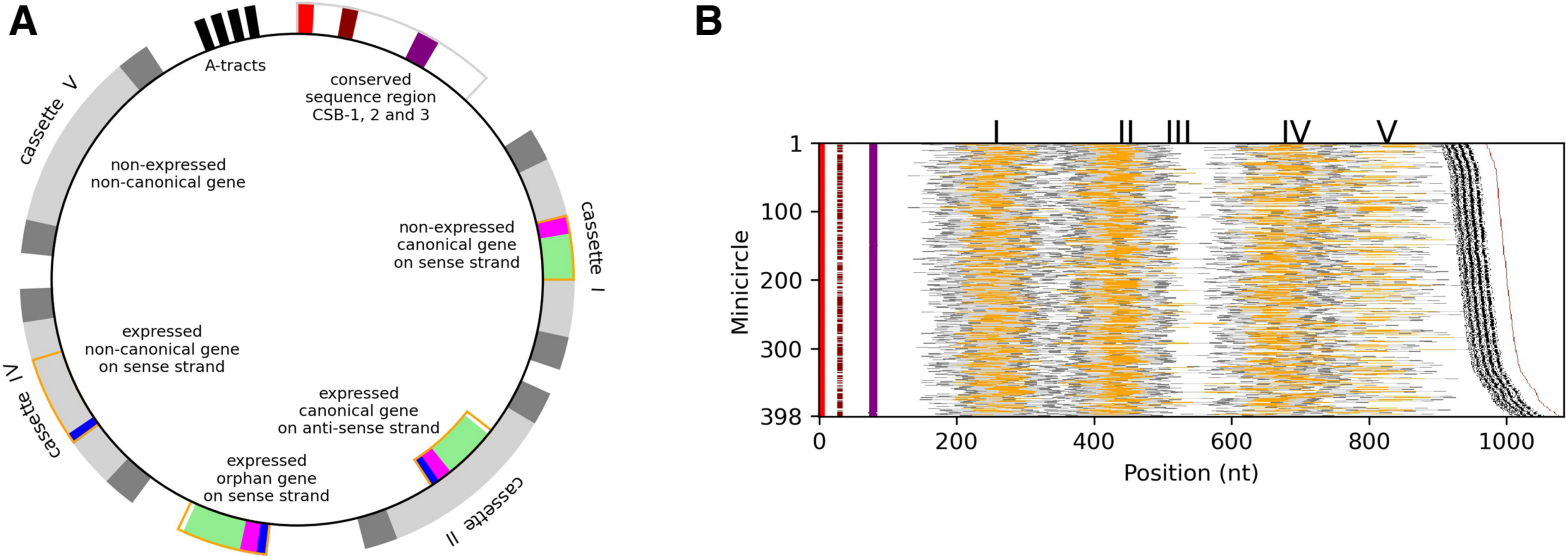
(*A*) Cartoon of a hypothetical *T. brucei* minicircle showing the typical positions of the major cassettes relative to the conserved sequence region (CSR). The CSR contains the highly conserved CSB-1 and CSB-3 motifs and the less well conserved CSB-2. Upstream of the CSR are four to five A-tracts, each ∼5 bp long and positioned roughly in phase with the helical repeat. The major cassettes are labeled (in 5′ to 3′ order) I, II, IV, and V and are on average 142 nt long. A minor cassette III exists between cassettes II and IV, on a few minicircles. Cassettes are defined by a forward and reverse pair of imperfect 18 bp repeats (dark gray segments). Many cassettes encode canonical gRNA genes that can identifiably align with edited mRNA. Guide RNAs are usually encoded on the sense strand (e.g., in cassettes I, IV, and orphan) or rarely on the antisense strand (e.g., in cassette II). Cassettes also encode noncanonical genes of unknown, if any, function that do not identifiably match edited mRNA without gaps (e.g., in cassettes IV and V). Canonical genes have an ∼11 nt anchor region (magenta segments) and a ∼31 nt guiding region (green segments) that direct U-insertion/deletion. Some gRNA genes are not encoded within a cassette. These so-called orphan gRNA genes have only been found between cassettes II and IV on the sense strand. Sequencing and alignment of small RNA transcripts allow determination of canonical and noncanonical gRNA gene expression status and identification of 5 nt transcription initiation sites (blue segments in cassettes II, IV, and the orphan). Initiation sequences are 30–32 nt downstream from the forward 18 bp repeat of sense strand encoded genes and 30–32 nt upstream of the reverse 18 bp repeat of antisense strand encoded genes. Gene extent (shown as orange rectangles) is either determined by alignment of transcripts for expressed genes (e.g., in cassettes II, IV, and orphan), or the complementarity to edited mRNA for nonexpressed canonical genes (e.g., in cassette I) or is not defined for nonexpressed noncanonical genes (e.g., in cassette V). (*B*) Structure of 398 assembled minicircles ordered by length (*rightmost* brown line). Magenta, brown, and purple represent conserved sequence blocks CSB-1, CSB-2, and CSB-3, respectively. A cassette is shown as light gray, with flanking 18 bp inverted repeats as dark gray. Cassette-associated and orphan expressed canonical and noncanonical gRNAs genes are shown in orange. The labels for the five gRNA cassette positions, I–V, are located at each cassette's median center position. Black shows A-tracts of the bend region. Reproduced from Cooper et al. (2019) with minor modifications.

The kDNA of *Trypanosoma brucei*, the causative agent of human sleeping sickness (human African trypanosomiasis) and African animal trypanosomiasis, is one of the most complex (Steinert and van Assel 1980). Early work hinted at between 300 to 400 minicircle classes (Stuart 1983). Guide RNA genes in *T. brucei* are usually encoded in ∼140 bp cassettes (Fig. 1A) which are framed by 18 bp imperfect inverted repeats (Jasmer and Stuart 1986; Pollard et al. 1990; Hong and Simpson 2003). Cassettes might be involved in gRNA expression or maturation (Suematsu et al. 2016; Cooper et al. 2019). Each minicircle class encodes three to four cassettes (Fig. 1A), suggesting that *T. brucei* might have over 900 gRNA genes with significant redundancy (Corell et al. 1993). Identification of a complete set of gRNAs, and their genes, that cover all canonical editing events in *T. brucei* (and other kinetoplastid species), has been a major goal in the field of U-indel editing. Recent deep sequencing of short mitochondrial RNAs in the procyclic (PCF) and bloodstream forms (BSF) of *T. brucei* EATRO 164 identified ∼210 gRNA populations that gave nearly complete coverage of the twelve known edited mRNAs (Koslowsky et al. 2014; Kirby et al. 2016). However, these studies faced the challenges of identifying gRNAs produced from the same versus a closely related gene and of distinguishing genuine gRNAs from other sequences. Assembly of minicircle classes overcomes these limitations by allowing unambiguous identification of gRNA genes within cassettes.

We recently reported the first essentially complete assembly and annotation of the minicircle genome from a differentiation-competent, laboratory-adapted strain of *T. brucei* (EATRO 1125 AnTat 1.1 90:13, here referred to as EATRO 1125; Engstler and Boshart 2004; Cooper et al. 2019). We were able to assemble and annotate 391 minicircle classes based on a 95% sequence identity threshold (Fig. 1B). Note that currently there is no generally accepted definition of a minicircle class. Minicircles with the same set of gRNAs can differ in sequence (Hong and Simpson 2003; Cooper et al. 2019), and conversely, closely related minicircle sequences can encode nonidentical sets of gRNA genes through genetic drift (Cooper et al. 2019). For this study, we have adopted the 95% sequence identity threshold definition from Cooper et al. (2019). All 391 assembled minicircles contained the ∼120 bp conserved sequence region (CSR) found in all kinetoplastids (Fig. 1), and which is thought to be involved in binding the minicircle replication machinery (Abu-Elneel et al. 1999; Shlomai 2004). This region includes the hyper-conserved sequence blocks (CSB) 1 and 3 and the less well conserved CSB-2 (Fig. 1; Ray 1989). Cooper et al. (2019) reported CSB-1 to be 100% conserved (GGGCGTGCA), and CSB-3 to have a major variant (GGGGTTGGTGTA) and a minor variant (GGGGTTGATGTA) in three minicircles. (Note the first 100 bp of the conserved sequence region, starting with the CSB-1 motif, were not counted toward the 95% identity threshold used for defining minicircle classes.) Just upstream of CSB-1 is a conserved region of four to five A-tracts (Fig. 1), each ∼5 bp long and positioned roughly in phase with the helical repeat. These cause a bend in the minicircle (Marini et al. 1982) that has been suggested to aid organization of minicircles into the kDNA structure (Jensen and Englund 2012). We confirmed that almost all minicircles encode three or four cassettes (Cooper et al. 2019).

We identified about 930 “canonical” gRNA genes (Fig. 1A), mostly in cassettes, by alignment to published edited mRNA sequences (allowing for some strain-specific polymorphisms). These covered nearly all known (i.e., canonical) editing events (accessible via http://hank.bio.ed.ac.uk). We also identified about 370 “noncanonical” gRNA genes (Fig. 1A) within cassettes of similar nucleotide frequency structure to canonical gRNA genes but of unknown function, if any. Most canonical gRNA genes were found to be encoded on the sense (or coding) strand, and eight were found on the antisense (or template) strand (Fig. 1A; Cooper et al. 2019). We also identified twelve “orphan” gRNA genes not encoded within cassettes between cassettes II and IV (Fig. 1A; Cooper et al. 2019).

We had used small-RNA transcriptome data from BSF and PCF parasites to assist gRNA gene annotation and confirm expression of the majority of canonical and noncanonical gRNA genes (Fig. 1A). The 5′ ends of the majority of transcripts mapping to sense strand mapped to a position 30–32 nt downstream from the forward 18 bp repeat (Cooper et al. 2019), consistent with earlier reports of a gRNA transcription initiation site (Pollard et al. 1990). Similarly for the eight gRNA genes encoded on the anti-sense strand, we found the 5′ ends of many transcripts mapping to a position 30–32 nt upstream (with respect to the sense strand) from the reverse 18 bp repeat (Cooper et al. 2019). The transcriptome also included many sense and antisense transcripts of lower abundance that predominantly mapped within cassettes (Suematsu et al. 2016; Cooper et al. 2019). Interestingly, their mapped 5′ ends had a normal distribution with a peak around 40 nt, suggesting distinct differences in the biogenesis of gRNAs (including those encoded in antisense orientation) and antisense short RNAs (Cooper et al. 2019).

These two data sets provide a rich resource to study the extent, evolution, and maintenance of kDNA complexity. The aim of the present study was to use these data sets to obtain a deeper understanding and a more precise definition of the cassettes and gRNA genes of *T. brucei*. In particular, we focus on identifying and examining differences between cassette-encoded canonical and noncanonical gRNAs: their structure, their sequences and their expression status, and the characteristics of their cassettes and their small RNA transcripts.

## RESULTS

### Structure of minicircles

Using our refined assembly method, we assembled and annotated 398 minicircle classes using a 95% sequence identity threshold. Eight of these are new minicircles that have very low copy numbers (data not shown) that the previous assembly had missed (Cooper et al. 2019). One of the original 391 minicircles was an incorrect assembly and has been removed (mO_321), and another was updated as it had a misaligned region (mO_261). The structures of all 398 minicircles are shown in Figure 1B. All but one of the new minicircles contain the canonical CSB-1 motif GGGCGTGCA. One of the new minicircles, mO_394, has an alternate CSB-1, GGGCGTGTA. As reported previously, all but three minicircles have the canonical CSB-3 motif GGGGTTGGTGTA (the other three have the alternative sequence GGGGTTGATGTA), and 288 minicircles contain the canonical version of CSB-2, TCCCGTGC. Our previous analysis identified four major gRNA gene cassette positions which are labeled I, II, IV, and V and are clearly visible in Figure 1B. A fifth minor position between positions II and IV, labeled III, was also identified. Only seventeen minicircles have cassette III. One minicircle has no cassettes and one minicircle has one cassette. The majority of minicircles (268, 67%) have three cassettes, 128 minicircles (32%) have four cassettes, and no minicircles have all five cassettes. As reported previously (Cooper et al. 2019), upstream of the conserved region that includes CSB-1, 2, and 3 is a region characterized by four to five A-tracts, each ∼5 bp long and positioned roughly in phase with the helical repeat that cause a bend in the DNA (Marini et al. 1982).

### Characteristics of gRNA gene cassettes

#### Association between gRNA gene type, expression status, and cassette position

In total, 1318 cassettes are identified (Supplemental File S1), 23 more than in Cooper et al. (2019). Of these, 931 encode either a single canonical gRNA (927 cassettes) or two canonical genes (four cassettes), and 387 encode noncanonical genes. Canonical gRNA genes are defined by a match to the set of published fully edited mRNA genes, corrected for strain-specific polymorphisms (Supplemental File S2; Cooper et al. 2019). Guide RNA coverage statistics for each mRNA species, including predicted number of missing gRNAs, are given in Supplemental File S3. Six canonical gRNA genes are encoded on the antisense strand. In addition, there are eleven “orphan” canonical gRNA genes (one less than in Cooper et al. (2019) due to the misassembled minicircle mO_321) which are not framed by 18 bp repeats and which all lie between cassettes II and IV (but not in cassette III), the single MURF2-2 gRNA gene in the maxicircle, and the COX2 gRNA gene, which is part of the COX2 mRNA's 3′ untranslated region.

Using pooled small-RNA transcriptome data from BSF and PCF cells (Cooper et al. 2019), we developed a novel statistical method to determine the expression status and position for each gRNA gene (see Materials and Methods for details). Here, a gene is defined as expressed when a statistically significant number of transcripts align at their 5′ ends within the transcription initiation region. This is defined as the region 30–32 nt downstream from the forward 18 bp repeat or, for the six gRNA genes encoded on the antisense strand, defined as the region 30–32 nt upstream of the reverse 18 bp repeat. An expressed gene's initiation site is defined as the site with the most number of transcript 5′ ends mapping to it; this site is always within the initiation region. An expressed gene's end position is defined as the 90th percentile position of the mapped 3′ ends of transcripts with an untemplated oligo(U)-tail (Supplemental Fig. S1). In total, 1214 (92%) gRNA genes are classified as expressed and 104 (8%) as nonexpressed (Table 2; Supplemental File S4). Of the canonical gRNA genes, 917 (98%) are expressed and only 14 (2%) are nonexpressed. This contrasts with noncanonical gRNA genes of which 297 (77%) are expressed and 90 (23%) are nonexpressed. There is, therefore, a significant association between gRNA type and expression ($\chi_{1}^{2}=174$, *P* < 0.001), with canonical gRNA genes more likely to be expressed than noncanonical gRNA genes.

**TABLE 2.**
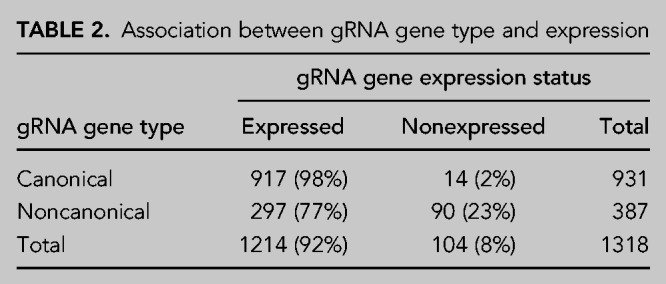
Association between gRNA gene type and expression

The type and expression of gRNA genes are not distributed evenly across cassette positions (Table 3; $\chi_{20}^{2}=97.3$, *P* < 0.001). In particular, cassette position I has relatively fewer expressed canonical genes (57%) than all other cassette positions combined (mean 75%), and relatively more nonexpressed noncanonical genes (16%) than all other cassette positions combined (mean 3%).

**TABLE 3.**
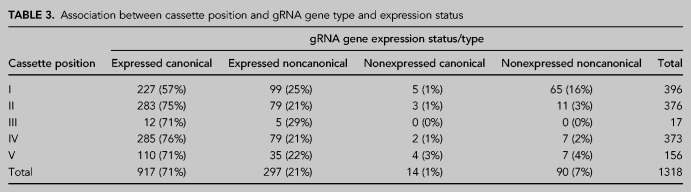
Association between cassette position and gRNA gene type and expression status

**FIGURE 2.**
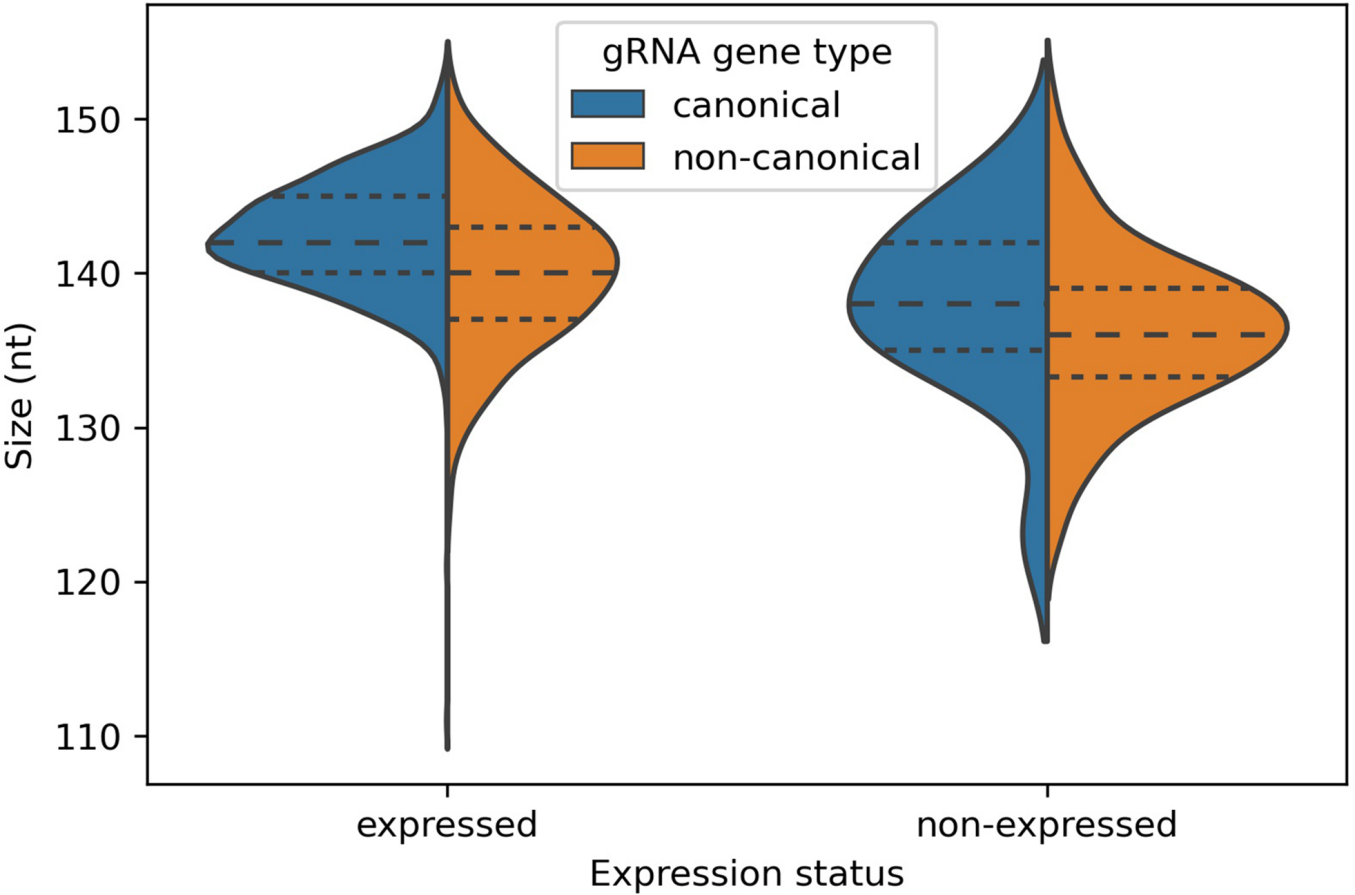
Association between cassette size (from 5′ end of forward 18 bp repeat to 3′ end of reverse 18 bp repeat) and gRNA type and expression status. Long-dashed lines: medians, short-dashed lines: first and third quartiles. Cassette sizes and standard deviations in brackets are as follows: expressed canonical: 142.6 nt (3.6 nt), expressed noncanonical: 140.2 nt (4.8 nt), nonexpressed canonical: 138.1 nt (5.8 nt), and nonexpressed noncanonical 136.4 nt (5.1 nt).

A plot of cassette size distributions (from the 5′ end of the forward 18 bp repeat to the 3′ end of the reverse 18 bp repeat) reveals differences between gRNA type and expression (Fig. 2). The mean size of cassettes encoding expressed canonical genes is 142.6 nt with a standard deviation of 3.6 nt. The size of cassettes encoding expressed canonical genes is significantly less variable (Levene test for equal variance, *F*_(3,1314)_ = 15.7, *P* < 0.001) and significantly larger (Kruskal–Wallis test, $\chi_{3}^{2}=170$, *P* < 0.001) than for cassettes encoding noncanonical and nonexpressed gRNA genes. Cassettes encoding canonical gRNA genes are, on average, 2.3 nt longer than cassettes encoding noncanonical gRNA genes (*t* = 8.5, *P* < 0.001). Cassettes encoding expressed gRNA genes are, on average, 3.7 nt longer than cassettes encoding nonexpressed gRNA genes (*t* = 8.3, *P* < 0.001).

The shorter lengths of cassettes encoding noncanonical and nonexpressed gRNA genes is because their genes are shorter. Expressed canonical gRNA genes have an average length of 48.6 nt, whereas expressed noncanonical genes are shorter by 2.9 nt. Expressed canonical gRNA genes have an average complementarity length (anchor region plus guiding region) of 40.4 nt, whereas nonexpressed canonical gRNA genes are shorter by 6.9 nt. (The gene lengths of nonexpressed canonical genes are not available because of insufficient transcripts mapping to them. Therefore, complementarity length is used rather than gene length when comparing nonexpressed canonical to expressed canonical genes.)

#### 18 bp inverted repeats and flanking regions

**FIGURE 3.**
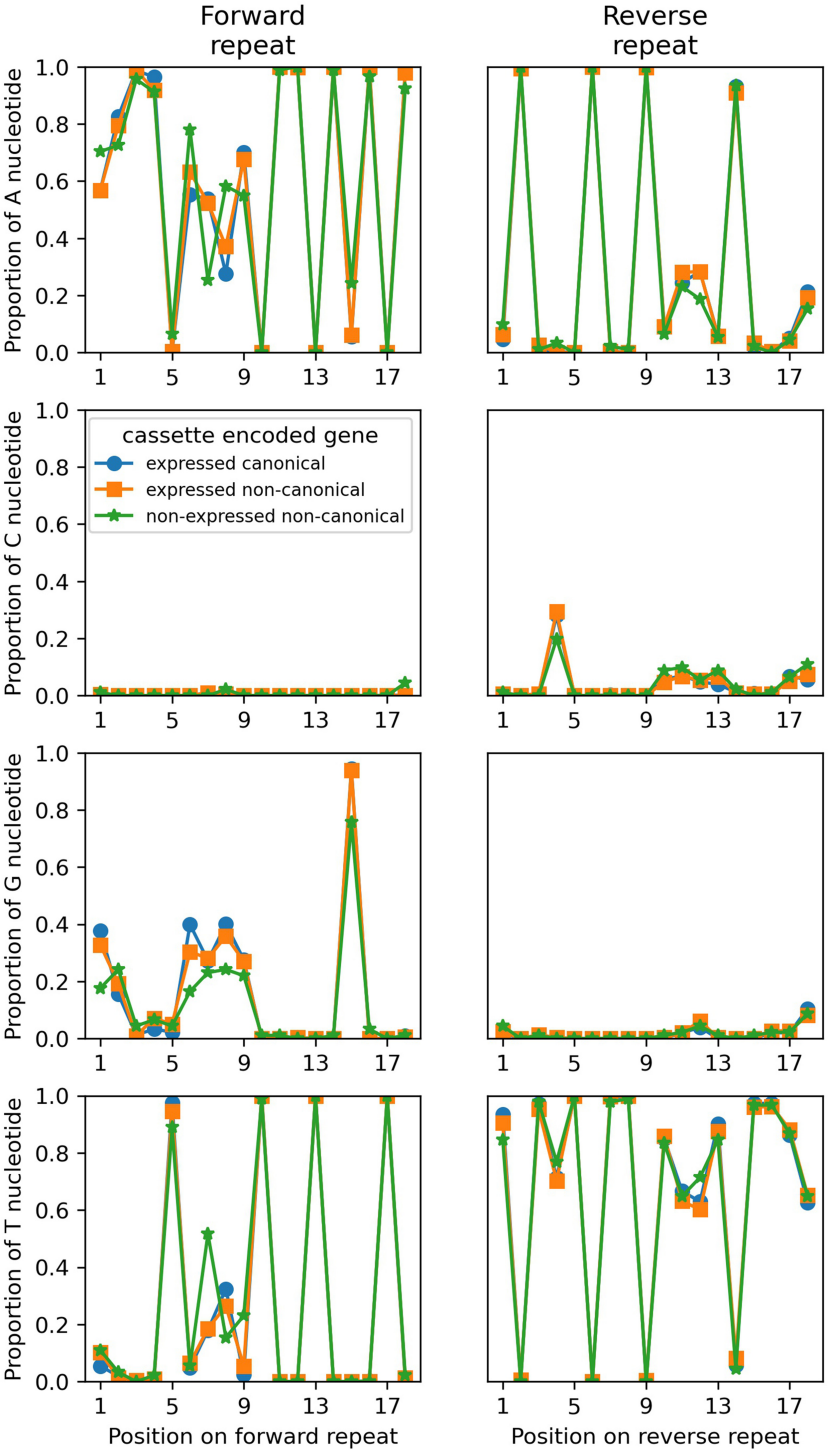
Nucleotide relative frequencies in the forward (*left* panels) and reverse (*right* panels) 18 bp inverted repeats for cassettes encoding expressed canonical gRNA genes (blue line with circles), expressed noncanonical genes (orange line with squares) and nonexpressed noncanonical genes (green line with stars). Expressed canonical genes are not shown due to their low number.

The sequences of the 18 bp inverted repeats were reported in Cooper et al. (2019). Nucleotide frequencies of the forward and reverse repeats are shown in Figure 3 (Supplemental Fig. S2). There is no significant difference of nucleotide frequencies of forward and reverse repeats between cassettes encoding expressed canonical (blue line with circles) and expressed noncanonical genes (orange lines with squares; forward: $\chi_{3}^{2}=1.28$, *P* = 0.73; reverse: $\chi_{3}^{2}=1.26$, *P* = 0.74). There is also no significant difference of nucleotide frequencies of the reverse repeat between cassettes encoding expressed noncanonical and nonexpressed noncanonical genes (green lines with asterisks; $\chi_{3}^{2}=0.63$, *P* = 0.89). However, there is a significant difference of nucleotide frequencies of the forward repeat between cassettes encoding expressed noncanonical and nonexpressed noncanonical genes ($\chi_{3}^{2}=16.6$, *P* < 0.001). This difference is mainly due to nucleotide frequency differences at positions 6, 7, 8, and 15 in the forward repeat (Fig. 3).

The forward and reverse repeats of each cassette are highly complementary (if allowing GU wobble in the corresponding transcripts). On average the number of complementary base pairs is 16.0 nt, with a standard deviation of 1.1 nt. Interestingly, the distribution of the number of complementary base pairs between randomly paired forward/reverse repeats is almost identical (mean 16.0 nt, st. dev. 1.1 nt to 1 decimal place). This perhaps suggests that forward and reverse repeats in transcripts from different cassettes may duplex, or it may reflect the requirement to interact with an external factor, such as an editing protein or protein complex (Cruz-Reyes et al. 2018; Aphasizheva et al. 2020), restricting sequence variation.

The number of complementary base pairs of cassettes encoding expressed canonical genes is slightly, but significantly, larger than for cassettes encoding noncanonical and nonexpressed gRNA genes (Kruskal–Wallis test, $\chi_{3}^{2}=27.3$, *P* < 0.001). Cassettes encoding expressed canonical gRNA genes have, on average, 0.2 and 0.5 more complementary base pairs in their repeats than cassettes encoding noncanonical genes (*t* = 2.28, *P* = 0.23) and nonexpressed genes (*t* = 4.04, *P* < 0.001), respectively. Whether these small differences are biologically significant remains to be shown.

**FIGURE 4.**
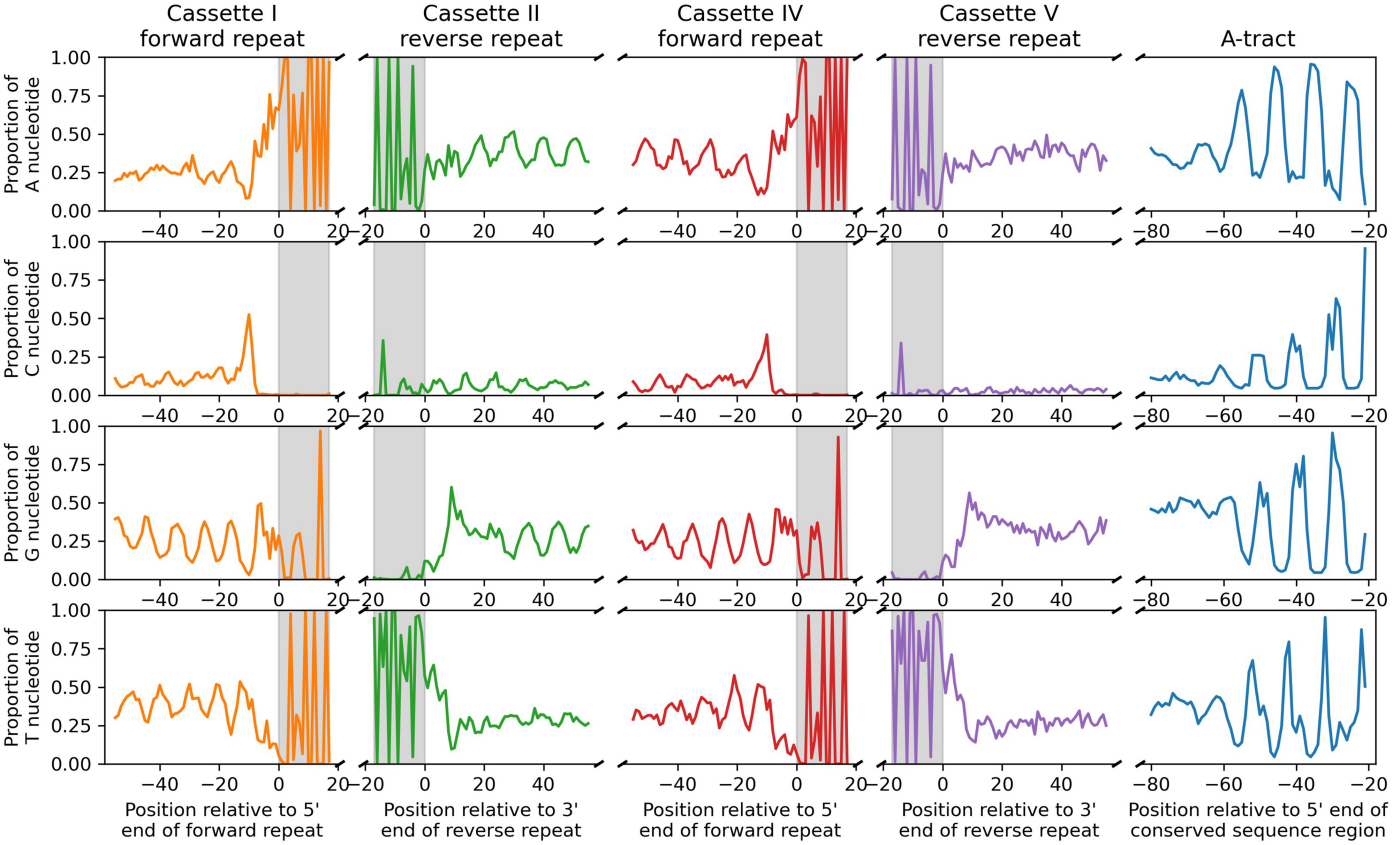
Nucleotide relative frequencies upstream of the forward 18 bp repeat of cassette I (orange), downstream from the reverse repeat of cassette II (green), upstream of the forward repeat of cassette IV (red), downstream from the reverse repeat of cassette V (purple), and the A-tract upstream of the CSR (blue). Oscillations in the nucleotide frequencies are roughly in phase with the helical repeat.

The major cassettes are structurally arranged in two pairs (Fig. 1B): Cassettes I and II are separated on average by 32.6 nt (st. dev. 21.9 nt), cassettes IV and V are separated on average by 19.2 nt (st. dev. 12.2 nt), but cassettes II and IV are separated on average by 110.2 nt (st. dev. 40.4 nt). The nucleotide frequencies flanking the cassettes exhibit interesting patterns (Fig. 4, gray regions represent the 18 bp repeats). Upstream of cassette I (Fig. 4, first column) are oscillations of G and T nucleotides in phase with the helical repeat. C nucleotide frequency exhibits a peak about 10 nt upstream of the forward repeat of cassette I. Downstream from cassette II (Fig. 4, second column) are 180° out-of-phase oscillations in A and G nucleotides and small oscillations in C nucleotides in phase with G nucleotides. There is also a matching peak in G nucleotides 10 nt downstream from the reverse repeat of cassette II. The nucleotide frequencies upstream of cassette IV (Fig. 4, third column) are similar to those upstream of cassette I with additional oscillations in A nucleotides. There are no oscillations downstream from cassette V (Fig. 4, fourth column), although there is a peak in G nucleotides 10 nt downstream from its reverse repeat. The functions of these oscillations are unknown. There is a similarity between the oscillations between cassettes II and IV to the oscillations upstream of the CSB-1 (Fig. 4, A-tract column) which cause a bend in the DNA (Marini et al. 1982). Perhaps the oscillations between cassettes II and IV cause another bend on the opposite side of the minicircle. It is not clear what the role of the oscillations upstream of cassettes I and IV could be.

#### Structure of cassettes

**FIGURE 5.**
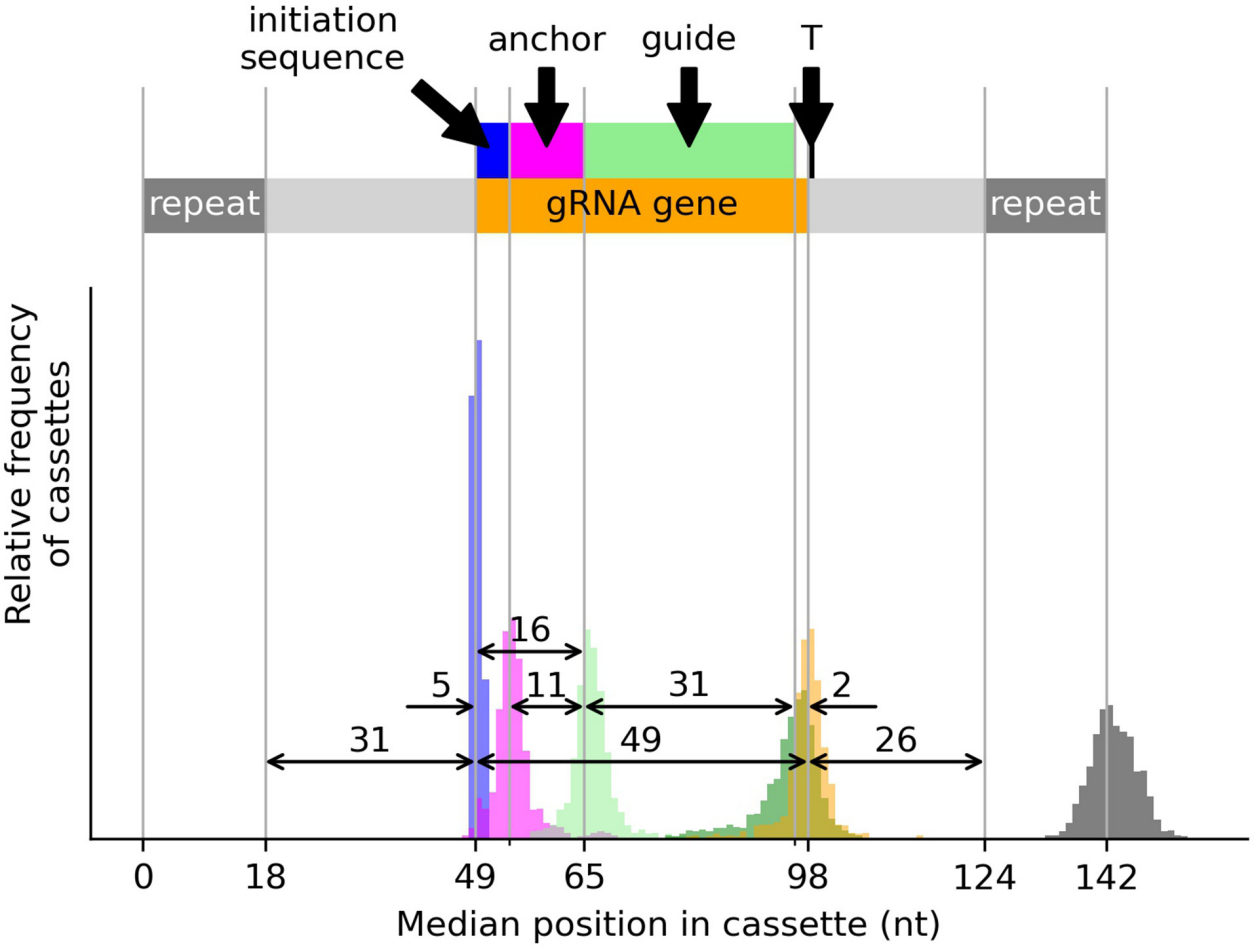
Structure of typical cassettes encoding typical expressed canonical gRNA genes aligned at the 5′ end of the 18 bp forward repeat (position 0 on *x*-axis). Histograms show the distributions of the positions of expressed canonical gRNA gene components relative to the 5′ end of the forward repeat: Blue: 5′ ends of 5 nt initiation sequences; magenta: 5′ ends of anchors; light green: 5′ ends of guiding regions; dark green: 3′ ends of guiding regions which typically ends 2 nt short of the gRNA gene; orange: 3′ ends of gRNA genes which are typically 49 nt long and commonly end with a T nucleotide; and dark gray: 3′ ends of 18 bp reverse repeats. The colored bars represent the positions of the gRNA gene components based on their median positions within cassettes. The double-headed arrows indicate median distances between cassette features.

Figure 5 shows the typical structure of cassettes encoding expressed canonical gRNA genes. The median length of these cassettes is 142 nt (gray histogram). The 5′ end of the 5 nt initiation sequence (represented as a blue rectangle) is positioned 30–32 nt (median 31 nt) from the 3′ end of the forward 18 bp repeat (blue histogram). The median gRNA gene length is 49 nt (distance between the medians of the blue and orange histograms, and defined as the distance from the 5′ end of the initiation sequence to the 90th percentile position of mapped 3′ ends of transcripts with untemplated oligo(U)-tails). The anchor (represented by the magenta rectangle) is typically positioned at the 3′ end of the initiation sequence but may overlap it. Its median length is 11 nt (magenta histogram). The guiding region (represented by the green rectangle) begins immediately after the anchor. Its median length is 31 nt (distance between the medians of the light and dark green histograms). The average number of editing sites (sites between non-T nucleotides) covered by guiding regions is 17.8 nt (st. dev. 4.5 nt). There is a median gap of 2 nt between the 3′ end of the guiding region and the 3′ end of the gene (distance between the medians of the dark green and orange histograms). The median gRNA gene complementarity length (anchor plus guiding region) is 42 nt (distance between the medians of the magenta and dark green histograms). The difference between the complementarity length (42 nt) and the gene length (49 nt) is due to the 5 nt initiation sequence and the two noncomplementary 3′ nucleotides.

### Characteristics of gRNA genes

#### Nucleotide frequency structure of gRNA genes

**FIGURE 6.**
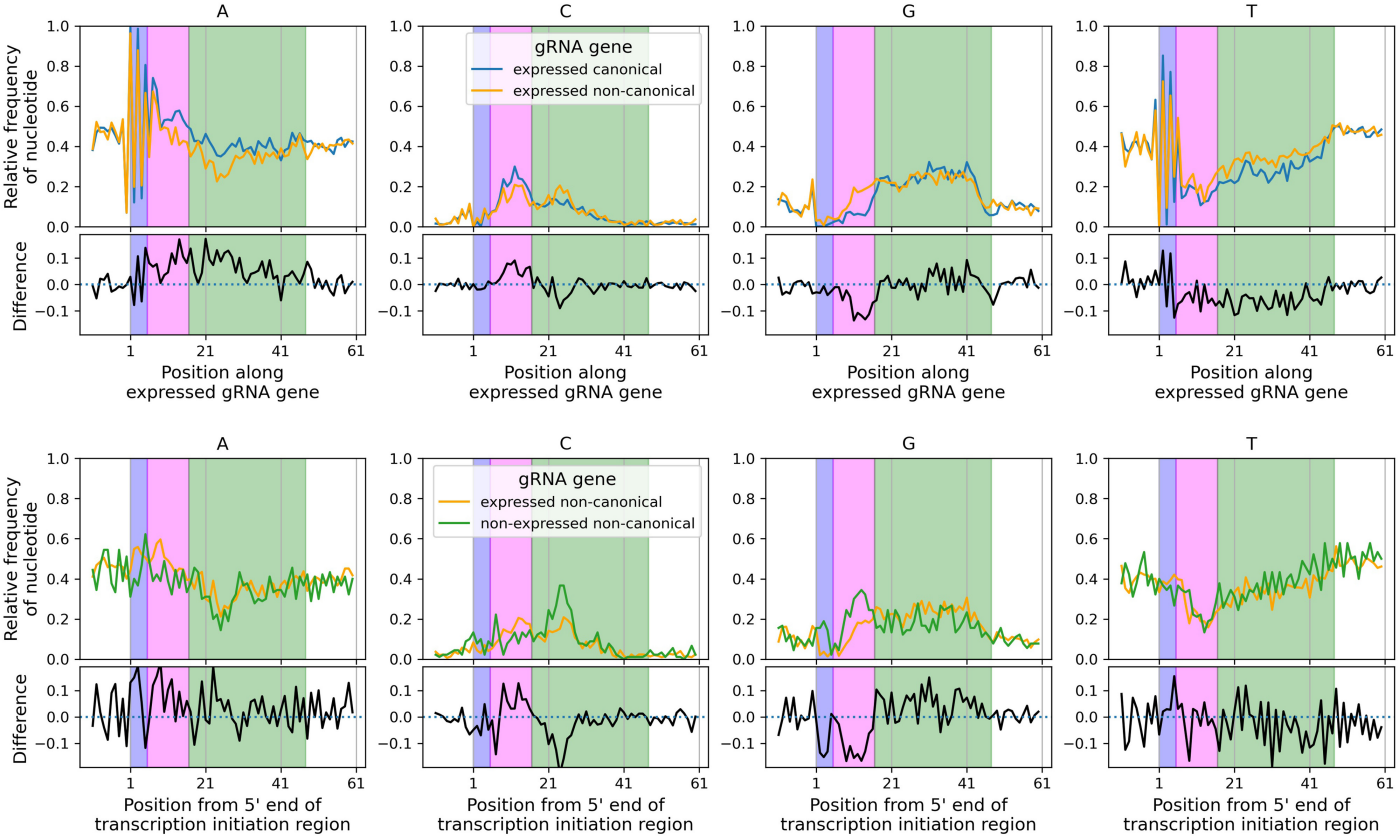
Nucleotide frequency structure of gRNA genes. (*Top* panels) Expressed canonical (blue lines) and expressed noncanonical (orange lines) gRNA gene sequences were aligned from 10 nt upstream of their 5 nt transcription initiation sequences (the first base of which is at position 1 on the *x*-axes) and the relative frequency of each nucleotide at each position was obtained. This reveals the ATATA initiation sequence (positions 1 to 5 highlighted by the light blue rectangle), the low-GT/high-AC region from positions 6 to 16 corresponding to the anchor region (light magenta rectangle, see Fig. 5), and the elevated G nucleotide frequency from positions 17 to 47 corresponding to the guiding region (light green rectangle). (*Bottom* panels) Nonexpressed noncanonical (green lines) and expressed noncanonical (orange lines) gRNA gene sequences were aligned from 10 nt upstream of the initiation region as nonexpressed genes do not have an initiation sequence. Subpanels with black lines show the differences in relative frequencies between expressed canonical and noncanonical genes (*upper* panels) and between expressed and nonexpressed noncanonical genes (*lower* panels).

Alignment of all cassette-associated, expressed canonical and noncanonical gRNA genes at the start of their initiation sequences reveals extensive similarities and interesting differences in nucleotide frequency structure (Fig. 6, top panels). For both categories of genes, the region from positions −10 to −1 are AT-rich and GC-poor. The region from positions 0 to 7, which encompasses the 5 nt initiation sequence, is characterized mainly by four consecutive TA-repeats. The region from positions 6 to 16 has a peak in C nucleotide frequency and a low G nucleotide frequency (more pronounced in canonical genes) and relatively low T nucleotide frequency. This region corresponds to the gRNA anchor, and these nucleotide frequencies reflect selection for Watson–Crick base pairs and selection against GU base pairs, presumably for stabilizing the gRNA–mRNA duplex at the anchor. In the region from positions 17 to 43, A nucleotide frequency remains constant at about 40% in canonical gRNA genes but is lower and more variable in noncanonical genes, C nucleotide frequency is elevated but declines gradually to about 2%, G nucleotide frequency remains elevated at about 25%, and T nucleotide frequency gradually rises from about 20% to 30%. This region corresponds to the gRNA guiding region in which G and A nucleotides mainly guide U-insertion into the mRNA. In the region after position 43, G nucleotide frequency abruptly declines to about 10%, and T nucleotide frequency abruptly increases to about 50%, reflecting the end of gRNA genes.

Although expressed canonical and noncanonical gRNA genes follow roughly the same pattern in their nucleotide frequencies, there are some interesting and significant differences. Nucleotide frequencies are significantly different in the region from positions 0 to 7 ($\chi_{3}^{2}=30.3$, *P* < 0.001) with peaks in T and A nucleotides being smaller for noncanonical than for canonical gRNA genes. The frequencies of C and G nucleotides in this region are similar between the two gene types, therefore, the smaller peaks imply that the alternation of T and A in noncanonical gRNA genes is less well conserved. Nucleotide frequencies are significantly different in the anchor region from positions 6 to 16 ($\chi_{3}^{2}=279$, *P* < 0.001). In particular, the elevated G nucleotide frequency in noncanonical gRNA genes suggests a weakened, or even absence of, selection for Watson–Crick base pairs in the gRNA–mRNA anchor duplex (Kirby et al. 2016). In noncanonical genes, there is an unusual C nucleotide peak in the region from positions 23 to 26 ($\chi_{3}^{2}=60.9$, *P* < 0.001) which corresponds to a drop in A nucleotide frequency. We have no explanation for this. Nucleotide frequencies are significantly different across the guiding region from positions 17 to 43 ($\chi_{3}^{2}=172$, *P* < 0.001) with A nucleotide frequency generally higher, and T nucleotide frequency generally lower, in canonical than noncanonical genes. After position 43 there is no significant difference between nucleotide frequencies ($\chi_{3}^{2}=6.1$, *P* = 0.11).

It is instructive to also examine nucleotide frequency structure in expressed and nonexpressed noncanonical gRNAs genes. Unlike expressed genes, nonexpressed genes do not have a transcription initiation site. This means we cannot align expressed and nonexpressed noncanonical genes at their initiation sites. Instead, we align these genes from the start of the transcription initiation region 30 nt downstream from the forward repeat (Fig. 6, lower panels). A consequence of this is a smoothing out of the TA-repeat peaks because the alignments of the ATATA initiation sequences are offset from each other. Examination of the differences in nucleotide frequencies reveals two regions of interest. Nucleotide frequencies are significantly different in the anchor region from positions 6 to 16 ($\chi_{3}^{2}=69.5$, *P* < 0.001). In particular, there is a higher G nucleotide frequency in nonexpressed noncanonical genes, perhaps reflecting a longer period of weakened or absent selection pressure for Watson–Crick base-pairing in the anchor. Nucleotide frequencies are significantly different in the region from positions 21 to 26 ($\chi_{3}^{2}=38.2$, *P* < 0.001). In particular, there is an elevated C frequency. Again, we have no explanation for this.

#### Characteristics of initiation sequences

Based on previous characterizations of gRNA 5′ ends, a 5′-RYAYA motif (IUPAC codes: R = {A, G}; Y = {C, T}) had been suggested as a consensus for gRNA gene transcription initiation sequences in *T. brucei* (Pollard et al. 1990). Subsequently, a more comprehensive analysis of gRNA transcripts in *T. brucei* strain EATRO 164 suggested a 5′-ATATA consensus (Koslowsky et al. 2014), a sequence that we also found in ∼60% of gRNA transcripts from expressed canonical genes (Table 4). In addition, we found a non-A nucleotide in position 5 in about 20% of expressed canonical genes (top left panel of Fig. 6). A more representative consensus is 5′-AWAHH (W = {A, T}; H = {A, C, T}) present in 95% of expressed canonical gRNA genes. We also note that the nucleotide just upstream of the initiation sequence is very rarely an A nucleotide and in ∼60% of cases is a T nucleotide (top right panel of Fig. 6). This position could therefore be considered part of a 6 nt transcription initiation motif, B|AWAHH (B = {C, G or T}) present in 87% of expressed canonical gRNA genes.

**TABLE 4.**
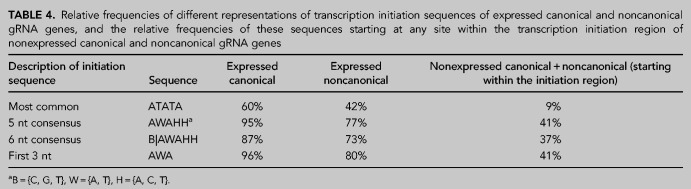
Relative frequencies of different representations of transcription initiation sequences of expressed canonical and noncanonical gRNA genes, and the relative frequencies of these sequences starting at any site within the transcription initiation region of nonexpressed canonical and noncanonical gRNA genes

As pointed out above, 60% of expressed canonical gRNA genes begin with an ATATA sequence, but only 42% of expressed noncanonical gRNA genes have this sequence (Table 4). This lower percentage reflects the greater diversity of initiation sequences of expressed noncanonical gRNA genes (53 different sequences) compared to expressed canonical gRNA genes (40 different sequences). In addition, 96% of expressed canonical gRNA genes have either ATA or AAA as their first three nucleotides of their initiation sequence, whereas only 80% of expressed noncanonical gRNA genes have initiation sequences starting with any ATA or AAA. Of the 104 nonexpressed gRNA genes (canonical and noncanonical) only 9% have ATATA, and only 41% have either ATA or AAA, starting anywhere within their initiation regions.

#### Characteristics of anchors

**FIGURE 7.**
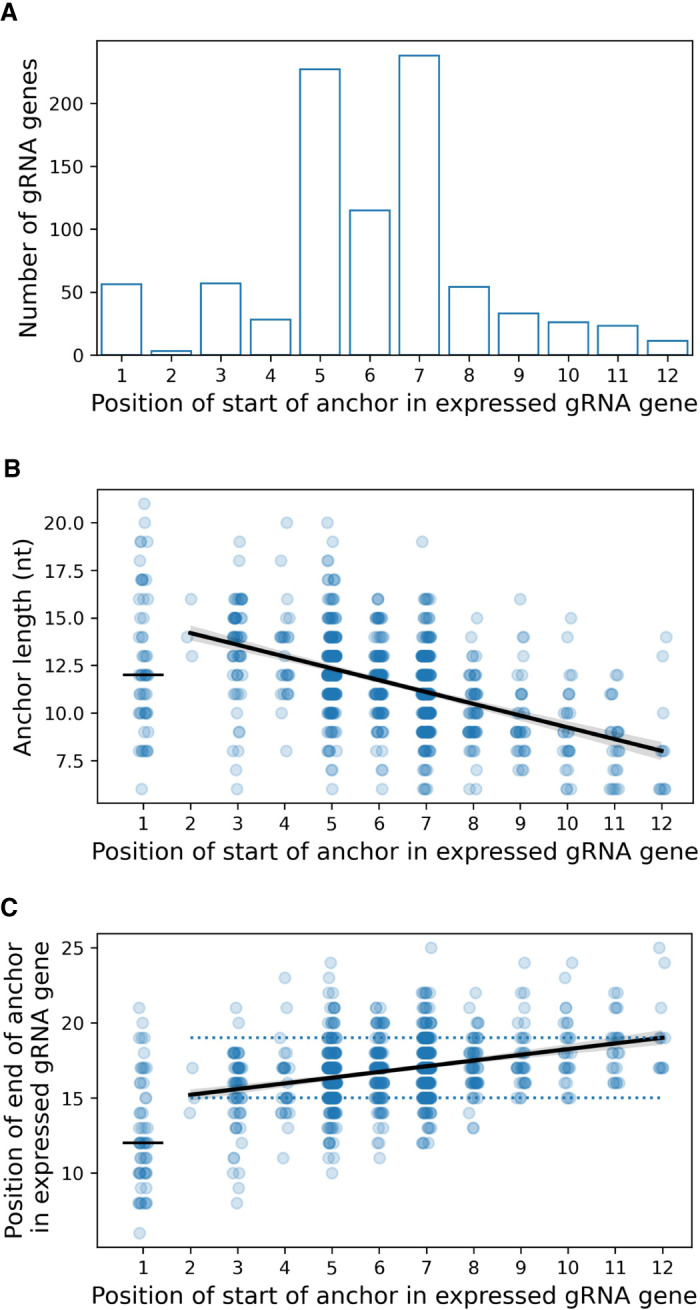
(*A*) Distribution of the position of the 5′ end of the anchor along cassette-associated expressed canonical gRNA genes. Position 1 is the 5′ end of the 5 nt initiation sequence (i.e., anchor completely overlaps initiation sequence). Anchors of most gRNA genes overlap the initiation sequence by 1 nt (position 5) or are separated from the initiation sequence by 1 nt (position 7). (*B*) For genes whose anchors do not completely overlap the initiation sequence (position ≥ 2), mean anchor length reduces by 0.6 nt (95%CI 5.4–6.9 nt) for each nucleotide separating the anchor from the start of the gene (*t* = 16.0, *P* < 0.001). (*C*) Also for these genes, the 3′ end position of an anchor is weakly correlated with its 5′ start position (*R*^2^ = 0.1). Guide RNA genes whose anchors completely overlap the initiation sequence (position 1) do not follow this pattern. Circles: data for individual genes with jitter added to separate circles; black lines: linear regression lines; dotted lines in *C*: 70% of genes have 3′ anchor positions between 15 and 19 nt from the start of the gene.

The gRNA anchor is defined as the longest, 5′-most, contiguous region of Watson–Crick complementarity to the cognate mRNA (Sturm and Simpson 1990). The majority of anchors begin 5 to 7 nt along cassette-associated expressed canonical gRNA genes (Fig. 7A; see also Fig. 4A in Koslowsky et al. 2014), either overlapping the 5 nt initiation sequence by 1 nucleotide (position 5 in Fig. 7A) or separated by 1 nt from the initiation sequence (position 7 in Fig. 7A). The mean anchor length is 11.4 nt with a range from 6 (the minimum cutoff of our canonical gRNA gene calling criteria) to 21 nt (Fig. 5). For gRNA genes with anchors not completely overlapping the initiation sequence (position ≥ 2 in Fig. 7B), mean anchor length shortens by 0.62 nt (95% CI 5.4–6.9 nt) for each additional nucleotide separating the anchor from the start of the gene (regression line in Fig. 7B, *t* = 16.0, *P* < 0.001). This means that the 3′ position of an anchor is only weakly correlated with its 5′ position (Fig. 7C; *R*^2^ = 0.1). As a consequence of the progressive shortening of anchor length with increasing distance of an anchor from the 5′ end of the gene, in the majority (70%) of such genes the 3′ positions of the anchors lie in a narrow range from 15 to 19 nt (median 17 nt) downstream from the start of the gene (Fig. 7C). Interestingly, the mean anchor length and 3′ position of gRNA genes completely overlapping the initiation sequence (short horizontal black lines at position 1 in Fig. 5B,C) do not fit this pattern.

**FIGURE 8.**
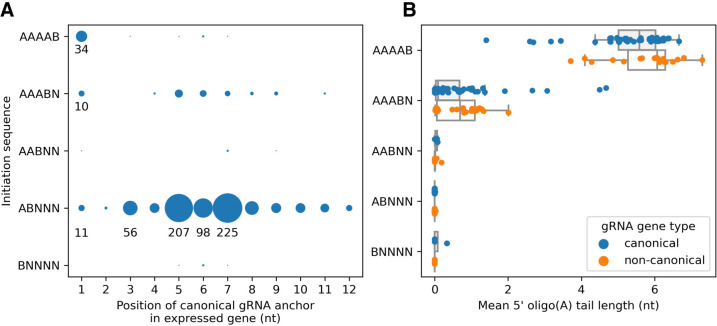
(*A*) Association between the 5 nt transcription initiation sequence and the position of the anchor in cassette-associated expressed canonical gRNA genes. IUPAC codes: B = {C, G, T}, N = {A, C, G, T}. Position 1 corresponds to complete overlap of the anchor with the initiation sequence. The number of genes, represented by the size of the circles, is indicated for some cases. (*B*) Association between the 5 nt transcription initiation sequence and the mean 5′ oligo(A) tail length of transcripts whose 5′ ends map to a cassette's initiation site for expressed canonical (blue) and noncanonical (orange) genes. Box plots: box represents the interquartile range (IQR), mid-line represents the median, whiskers represent 1.5 × IQR.

There is a significant association between the position of anchors in cassette-associated expressed canonical gRNA genes and their initiation sequences (Fig. 8A, $\chi_{96}^{2}=528$, *P* < 0.001). Thirty-nine of these genes have the A-rich AAAAB initiation sequence (IUPAC code: B = {C, G, T}) and 34 (87%) of these have anchors that completely overlap their initiation sequences (position 1, Fig. 8A). This is the case for only 10 of 66 genes (15%) with initiation sequence AAABN and 11 of 805 genes (1%) with initiation sequence ABNNN (which includes the most common sequence ATATA).

#### Orphan gRNA genes

Orphan gRNA genes are canonical genes that are not framed by an upstream 18 bp forward repeat and a downstream 18 bp reverse repeat. The eleven orphan gRNA genes lie between cassettes II and IV (but not in cassette III), all on different minicircles. These are not false positives because at least nine are expressed and their transcripts uniquely edit regions of CYb (6 genes), ND7 (2 genes), and CR4 (3 genes). In fact, all CYb gRNAs are encoded as orphan genes (Riley et al. 1994; Cooper et al. 2019). These genes appear to have no common initiation region relative to the upstream 18 bp repeat of cassette II. Mapping their associated transcripts to the minicircle sequences reveals that six of them have initiation sequences AAAAY (IUPAC code: Y = {C, T}), and one each have sequences ATAAC, ATATC, and AATAA; two genes have too few transcripts to consider them expressed. Hence, the initiation sequences of most orphan genes are still consistent with the AWAHH consensus identified above.

#### Characteristics of pooled BSF and PCF small RNA transcripts

##### Nontemplated 3′ oligo(U) tails

**FIGURE 9.**
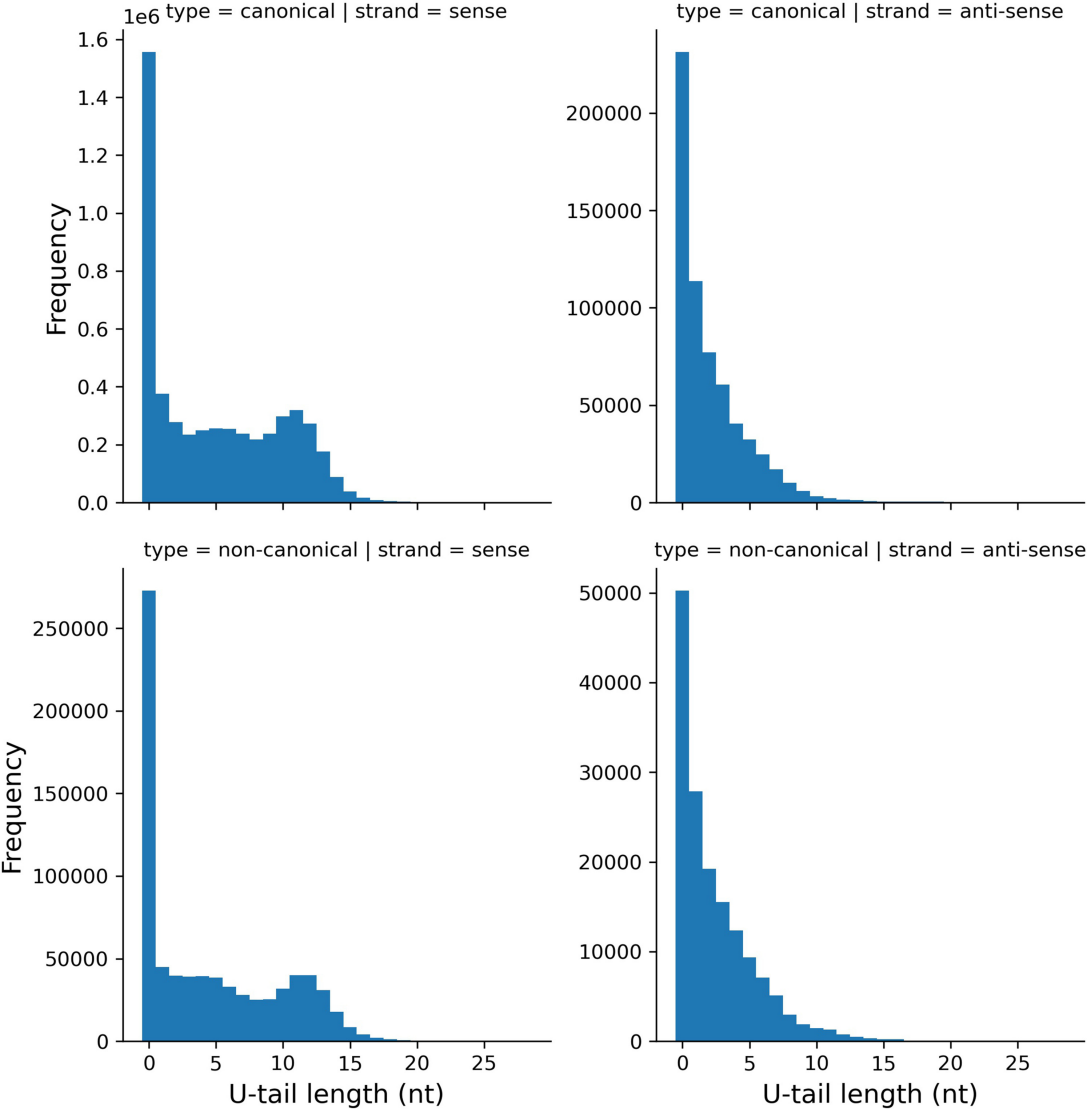
Distribution of oligo(U) tail lengths of transcripts mapping to cassettes encoding canonical (*top* row) and noncanonical (*bottom* row) gRNA genes and mapping to the sense (*left* column) and antisense (*right* column) strands. A transcript without an oligo(U) tail is recorded as having a U-tail length of zero.

Nontemplated 3′ oligo(U) tails are present on 70% of transcripts mapping to the sense strand of cassettes encoding canonical gRNA genes and 64% of transcripts mapping to the sense strand of cassettes encoding noncanonical gRNA genes (Fig. 9, left-hand panels; U-tail length ≥ 1). Other than that the distributions are similar (compare top left and bottom left panels). Many small transcripts also map to the antisense strand of cassettes (Cooper et al. 2019). U-tails are present on 64% of them (Fig. 9, right-hand panels; U-tail length ≥ 1). The distributions of U-tail lengths differ markedly between transcripts mapping to sense and antisense strands (compare left and right panels). On average, transcripts with U-tails mapping to the sense strand have an average U-tail length of 7.2 nt; this compares to 3.5 nt for transcripts with U-tails mapping to the antisense strand.

The distributions in Figure 9 are similar to the ones reported for *T. brucei* strain Lister 427 (Suematsu et al. 2016). A notable difference was that we found a much higher proportion of gRNAs lacking nontemplated oligo(U) tails. This can be partially explained as follows: 59% of the gRNAs that lack nontemplated U nucleotides added by terminal uridylylation end in a U nucleotide added by templated transcription. Suematsu et al. (2016), lacking information about templated versus nontemplated terminal nucleotides, presumably counted these molecules as uridylylated gRNAs.

**FIGURE 10.**
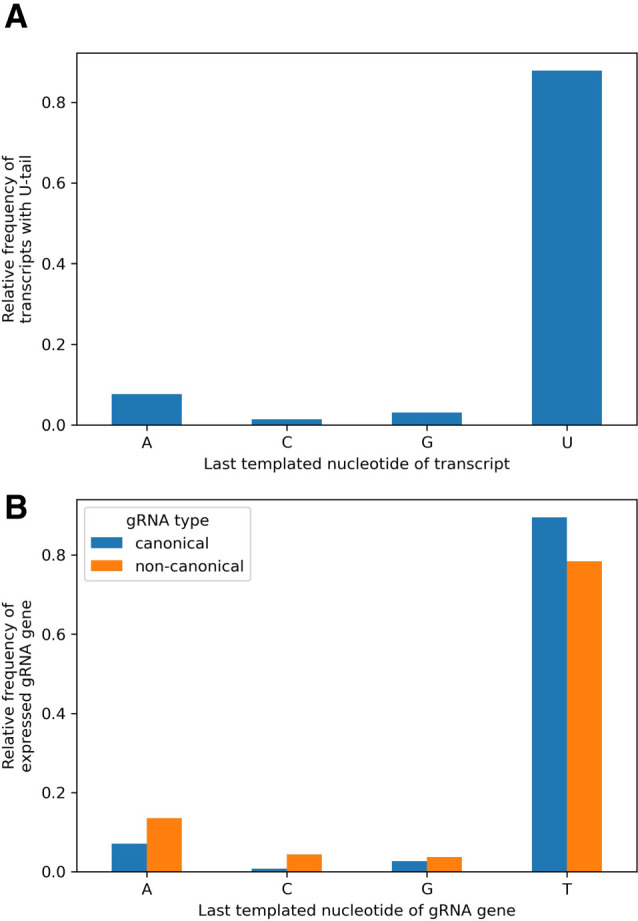
(*A*) Oligo(U) tails are attached preferentially to sense strand transcripts whose last (3′-most) templated nucleotide is U (canonical and canonical gRNA genes). (*B*) The last nucleotide of expressed canonical and noncanonical gRNA genes is usually T, but the proportion of genes ending with a T nucleotide is significantly lower for noncanonical gRNA genes.

Oligo(U) tails are attached preferentially to sense strand transcripts whose last templated nucleotide is a U (Fig. 10A). This perhaps reflects the preference of the RET1 TUTase enzyme for substrates ending in that nucleotide (Aphasizhev and Aphasizheva 2008; Aphasizheva and Aphasizhev 2010). Interestingly, relatively more expressed canonical gRNA genes (90%, Fig. 10B) terminate with a T nucleotide than expressed noncanonical gRNA genes (78%; $\chi_{1}^{2}=33.1$, *P* < 0.001). This probably explains why fewer transcripts mapping to noncanonical genes have oligo(U) tails.

##### Nontemplated 5′ oligo(A) tails

Post-transcriptionally added 5′ oligo(A) tails are associated mainly with gRNAs whose initiation sequences start with 3 or 4 A nucleotides (Fig. 8B). Mean oligo(A) tail lengths vary from about 2 to 7 nt (mean 5.4 nt) for genes whose initiation sequences start with four A's and vary from 0 to 5 nt (mean 0.6 nt) for genes whose initiation sequences start with three A's. Genes whose initiation sequences start with zero, one or two A nucleotides rarely have an oligo(A) tail. This finding supports the notion (Koslowsky et al. 2014) that these additional A nucleotides could result from RNA polymerase slippage events, as observed for certain viruses and bacteria (Volchkov et al. 1995; Lin et al. 2015). The nontemplated A nucleotides extend the anchor sequence by up to 8 nt in some cases, but on average by only 2.6 nt. This led us to speculate that some noncanonical gRNA genes with A-rich initiation sequences may have been misclassified due to our criteria for canonical gRNA genes of a minimum encoded anchor of 6 Watson–Crick base pairs. However, an analysis of such gRNA genes (31 in total) did not reclassify any of these genes as canonical.

It is worth noting that it is the genes whose anchors completely overlap with their initiation sequences that have the following three unique properties: (i) A-rich initiation sequences (position 1 in Fig. 8A), (ii) anchors that end closer to the start of their genes (position 1 in Fig. 7C), and (iii) transcripts with 5′ oligo(A) tails. We will discuss this interesting observation later.

##### Templated region of gRNAs

**FIGURE 11.**
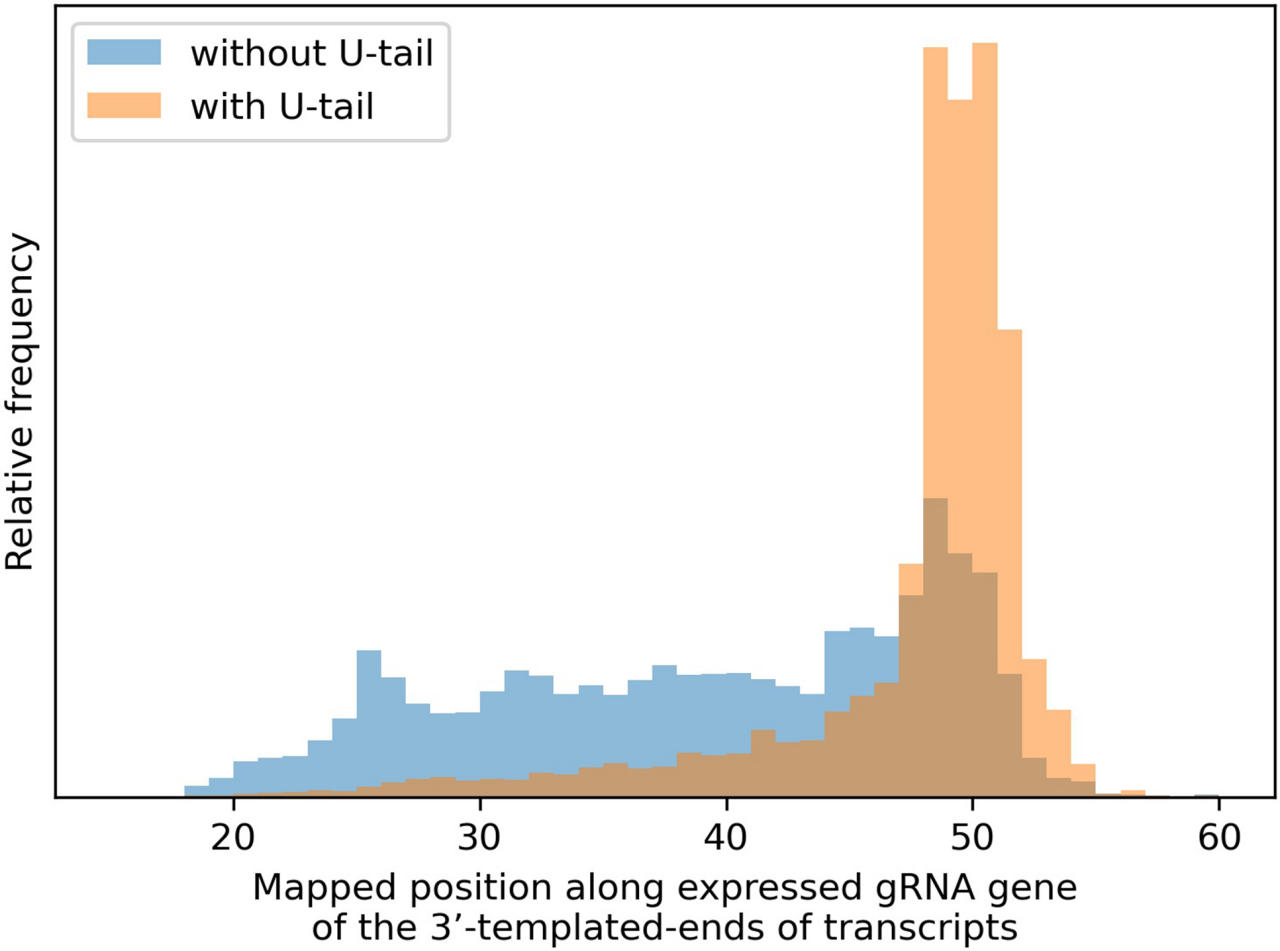
The distributions of where the templated 3′ ends of transcripts map with respect to the 5′ end of expressed gRNA genes (canonical and noncanonical) for transcripts with (orange) and without (blue) oligo(U) tails.

The distributions of where the templated 3′ ends of transcripts map with respect to the 5′ end of expressed gRNA genes is shown in Figure 11. Transcripts with a U-tail have a well-defined peak around positions 48 to 50 nt (this is similar for canonical and noncanonical genes; data not shown). In contrast, transcripts without U-tails have a much broader distribution. We do not know whether this reflects earlier termination of transcription or 3′ degradation of transcripts.

## DISCUSSION

We recently reported the first ever deep-sequencing and almost complete assembly of the kinetoplast DNA of a differentiation-competent strain of *T. brucei* (EATRO 1125 AnTat1.1 90:13; Cooper et al. 2019). In addition, and to confirm expression of gRNA genes, we also sequenced the kinetoplast's small RNA transcriptome in the same cell line in BSF and PCF parasites (Cooper et al. 2019). These two data sets provide a rich resource to study the extent, evolution and maintenance of kDNA complexity. The aim of the present paper is to use these data sets to obtain a deeper understanding and a more precise definition and description of gRNA genes and their transcripts. In the process, we improved upon (i) our previous minicircle assembly by using a dedicated kDNA assembler package (KOMICs, Van den Broeck et al. 2020), (ii) our gRNA gene identification pipeline, and (iii) the classification of expressed gRNA genes and their positions on minicircles with a more consistent statistical based approach using small RNA transcriptomics. We performed statistical analyses of the putative association of the characteristics of gRNA genes (i.e., type, length, encoding cassette position and size, flanking 18 bp repeats and initiation sequences) with their expression status. This provided a clearer and more precise description of the structure and sequence of typical gRNA genes. Finally, we analyzed the characteristics of the untemplated 5′ oligo(A) and 3′ oligo(U) tails of gRNA transcripts.

A schematic of the typical structure of an expressed canonical gRNA gene within a cassette framed by flanking 18 bp inverted repeats is shown in Figure 5 with median distances between features indicated. Our definition of canonical gRNA genes depends to some extent on the arbitrary cutoffs we use to call them (such as a minimum anchor length and a maximum number of mismatches). This probably means we misclassify some genes whose criteria lie close to these cutoffs. But, given the strong associations we see between gene characteristics, these misclassifications are unlikely to overly influence our conclusions.

**FIGURE 12.**
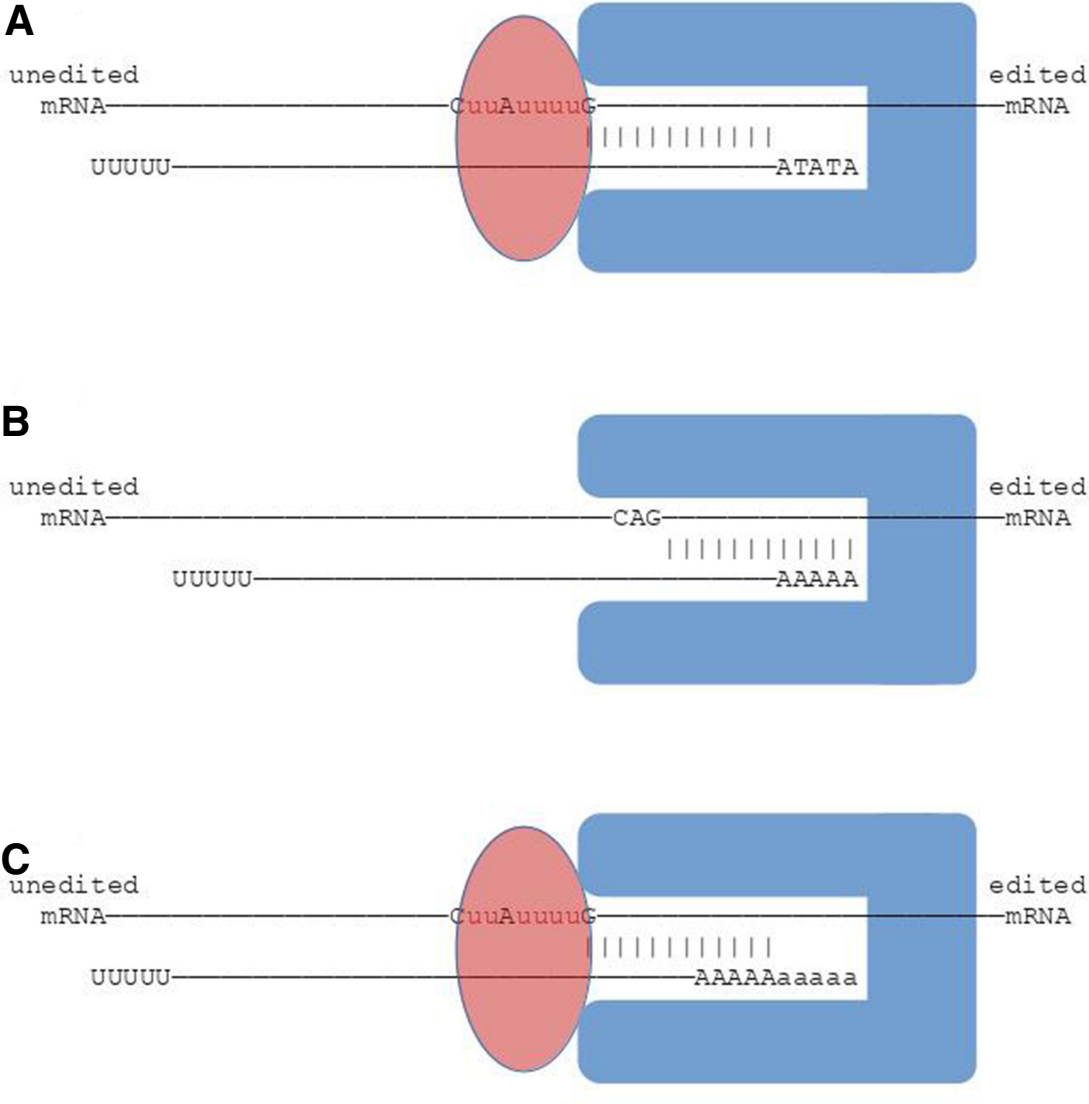
Schematic of a hypothesized molecular ruler that directs editing. (*A*) Guide RNAs with the ATATA initiation sequence followed by an 11–12 nt anchor fit just inside the molecular ruler (blue shape), which allows editing of editing sites (“u”s in the magenta ellipse). (*B*) The 5′ end of anchors of gRNAs whose anchors completely overlap their initiation sequences (almost always AAAAB or AAABN) would be buried within the ruler, thereby preventing editing of editing sites (e.g., between C, A, and G on the mRNA). (*C*) This is compensated for by the ∼5 nt oligo(A) tails that are attached to such gRNAs (Fig. 8B).

The 5′ end of a gRNA gene begins at the transcription initiation site located within the transcription initiation region which lies 30–32 nt downstream from the 3′ end of the forward repeat (Pollard et al. 1990). The 5′ end of 94% of small RNA transcripts mapping to the sense (or coding) strand within cassettes align to this region (Cooper et al. 2019). The 5 nt transcription initiation sequence is associated with the position of the gRNA anchor in the gene (Fig. 8A). If the anchor completely overlaps the initiation sequence (which occurs in 7% of genes), the most common initiation sequences are AAAAB or AAABN (IUPAC codes: B = {C, G or T}, N = {A, C, G, T}). Otherwise, the most common initiation sequence is ATATA. Most anchors overlap the initiation sequence by 1 nt or start 1 or 2 nt downstream from it (Fig. 7A). Ninety percent of gRNA genes have anchors 7–16 nt in length, with a median of 11 nt (Fig. 5), confirming earlier work by Kirby et al. (2016). Anchors are AC-rich and GT-poor which prevents GU wobble base-pairing with the mRNA (Fig. 6), and, therefore, presumably, helps to prevent nonspecific binding to mRNA. Interestingly, we discovered that the combined length of the initiation sequence and the anchor is remarkably conserved at 15–19 nt (Fig. 5, length of blue and magenta rectangles; and Fig 7C). Conceivably this could reflect the existence of a molecular ruler (Fig. 12A) with a role in deciding whether a gRNA should be accepted by the editing machinery or whether it should be rejected.

A molecular ruler may also explain the observation that the initiation sequences of genes whose anchor sequences completely overlap with their initiation sequences are usually AAAAB or AAABN rather than the more common ATATA initiation sequence. The anchor length of these genes is typically 12 nt (Fig. 7C) which would cause the first editing sites to be buried within the molecular ruler (Fig. 12B) preventing editing. However, the transcripts of these genes usually have nontemplated 5′ oligo(A) tails of mean length 5.4 nt that may result from RNA polymerase slippage events (Volchkov et al. 1995; Koslowsky et al. 2014; Lin et al. 2015). Such tails would push the anchor toward the editing machinery thus allowing editing to occur (Fig. 12C). In addition, most orphan genes begin with AAAA and their transcripts also have 5′ oligo(A) tails.

The guiding region is typically about 31 nt long (Fig. 5) and typically ends 2 nt short of the 3′ end of the expressed gene (Fig. 5; Supplemental Fig. S1). In other words, gRNA genes typically include two templated nucleotides between the guiding region and, if present, the U-tail, that do not match edited sequence. This finding confirms the observation of Koslowsky et al. (2014) of nonguiding nucleotides after the guiding region and before the U-tail in gRNA. Guide RNA genes most commonly end with a T nucleotide (Fig. 10B), presumably to facilitate addition of a U-tail (Aphasizhev and Aphasizheva 2008).

Almost a third of cassettes contain gRNA genes that do not align to canonically edited mRNA sufficiently well to identify their function; a value similar to that found by Corell et al. (1993) based on a ten times smaller data set that had been assembled from different strains and species. In line with the notion of “noncanonical” editing events (Simpson et al. 2016), we have called these genes “noncanonical” (Cooper et al. 2019). A few of them are unambiguously related to canonical genes as they can be aligned well to edited mRNA if gaps are allowed (Cooper et al. 2019). However, our experience is that allowing gaps usually results in many nonspecific alignments, making it impossible to identify a single obvious alignment for most noncanonical genes. The similar nucleotide frequency structure of noncanonical and canonical genes (Fig. 6, top panels) strongly suggests that the function of noncanonical genes is, or has been, the same as canonical genes. What is unclear is whether noncanonical genes are functionally important, or whether they are decaying remnants of once functional gRNA genes, or a mixture of both.

As argued in Cooper et al. (2019), the former hypothesis is controversial, despite accumulating evidence for the persistence of noncanonical editing patterns in mRNA populations (Ochsenreiter et al. 2008a; Kirby et al. 2016; Simpson et al. 2016; Gerasimov et al. 2018; Kumar et al. 2020) and evidence that at least one alternatively edited mRNA is translated into a protein, AEP-1 (Ochsenreiter and Hajduk 2006; Ochsenreiter et al. 2008b). An alternatively edited variant of the A6 mRNA has been documented for different strains, although in this case the predicted protein product is unchanged (Koslowsky et al. 2014; Madina et al. 2014; Cooper et al. 2019; Kumar et al. 2020). The two variants can be explained by the use of two different initiator gRNAs (Cooper et al. 2019). Clearly, far more evidence of alternative editing of mRNA would be required to explain the hundreds of noncanonical gRNA genes in *T. brucei*’s minicircles.

The evidence that noncanonical gRNA genes are decaying remnants of once functional gRNA genes is more compelling. Compared to canonical genes, noncanonical genes are less likely to be expressed (98% vs. 77%, Table 2) and those that are have fourfold lower transcript levels than canonical genes (Cooper et al. 2019; with the caveat that the correlation of functionality and transcript level is unclear, e.g., gRNAs may get consumed in the process of editing [Read et al. 2016]). The cassette sizes of noncanonical genes are 2.3 nt smaller and more variable (st. dev. of 5.1 nt vs. 3.7 nt, Fig. 2); the latter result perhaps suggesting loss of selection pressure to maintain a discrete size range. A certain distance between forward and reverse repeats may be important for efficient expression of the contained gRNA gene, for example, via binding of a dimeric transcription factor; a more relaxed distance could thus be mechanistically linked to the generally lower transcript levels that we observe. Noncanonical genes are less likely to have the common ATATA initiation sequence than canonical genes (42% vs. 60%, Table 4) and have more variable initiation sequences (53 vs. 40) even though they make up only 29% of all genes. They are less likely to have nontemplated oligo(U) tails (64% vs. 70%) and they are less likely to terminate with a T nucleotide (78% vs. 90%). Finally, although their nucleotide frequency structure is similar to canonical genes, their putative anchor regions contain more G nucleotides than canonical genes (Fig. 6, top panels). This is even more pronounced in nonexpressed noncanonical genes (Fig. 6, bottom panels). This suggests loss of selection pressure to maintain low G nucleotide frequency in the anchor for stabilizing the gRNA–mRNA duplex by preventing GU wobble base-pairing. Thus, the cumulative evidence points to the loss of function, and the consequent gradual decay by mutation, of the characteristics that define functional gRNA genes.

This hypothesis does not explain the sequence conservation of the inverted 18 bp repeats framing noncanonical gRNA genes. One might expect that, if a gene has lost its function and is accumulating mutations, its flanking 18 bp repeats would do so as well, but this is not what we found. Perhaps multiple pairs of 18 bp repeats are necessary for transcription of the long (∼600–800 nt) polycistronic precursor gRNAs (Grams et al. 2000; Aphasizheva and Aphasizhev 2010) or their processing into monocistronic gRNAs via secondary structure interactions with RET1 (Aphasizheva and Aphasizhev 2010). The existence of structure in the flanking regions of the two pairs of cassettes provides some tentative evidence for this (Fig. 4). Alternatively, there could be functional links between minicircle transcription and replication, as is the case for mammalian mtDNA replication (Gustafsson et al. 2016). This is speculative and more work needs to be done to understand the role of 18 bp repeats in gRNA transcription and processing.

The method of small RNA cloning and sequencing used by Cooper et al. (2019) does not allow us to definitively associate 5′ ends of transcripts with initiation sites. However, the fact that the vast majority of them do map to the initiation region 30–32 nt downstream from the forward 18 bp repeat (Pollard et al. 1990), and our discovery of a similar initiation region on the antisense strand of cassettes encoding gRNA genes on the antisense strand, suggest that the 5′ ends of the vast majority of sense strand transcripts, and some antisense strand transcripts, are indeed initiation sites. We propose new methods to classify gRNA gene expression and determine their positions within cassettes based on the statistical properties of the mapped 5′ and 3′ templated ends of small RNA transcripts. Our method of classifying expression uses the abundance of transcripts mapping to the initiation region relative to the background level of transcripts within a cassette. Consequently, a high transcript abundance within a cassette does not signify expression unless there are significantly more transcripts mapping to the initiation region than expected by random chance. It is encouraging that our expression classification correlates with many characteristics of cassettes and gRNA genes. Our method of defining the extent of gRNA genes within cassettes matches well with the characteristics of the templated 5′ and 3′ ends of gRNA transcripts discussed in Koslowsky et al. (2014).

We identified eleven “orphan” gRNA genes. Interestingly, they all lie between the reverse 18 bp repeat of cassette II and the forward 18 bp repeat of cassette IV. The complementarity between forward and reverse repeats suggests that either base-pairing between them, mediated by RNA secondary structure, or binding by a perhaps dimeric protein factor has some role in gRNA gene transcription (Pollard et al. 1990). The complementarity between the reverse repeat of cassette II and the forward repeat of cassette IV is just as high as between repeats within cassettes. In addition, many transcripts map to the antisense strand within cassettes (Figs. 7, 11; Suematsu et al. 2016; Cooper et al. 2019). Therefore, it is conceivable that repeats from neighboring cassettes can facilitate transcription of genes between them, perhaps akin to the mechanism that generates antisense transcripts within cassettes. Indeed, low levels of transcripts are found that map to the region between cassettes II and IV on many minicircles (Cooper et al. 2019). Clearly, more work needs to be done to understand the precise role of the 18 bp repeats in transcription.

We discovered two characteristics of gRNA genes for which we presently have no explanation. (i) Guide RNA genes in cassette position I being more likely to be nonexpressed noncanonical, and less likely to be expressed canonical than other cassette positions (Table 3); (ii) an elevated C frequency and depressed A frequency at the 5′ end of the guiding region in noncanonical gRNA genes (Fig. 6). Hopefully, future analyses of complete minicircle repertoire assemblies of other species and structural analysis of editing complexes in association with mRNA and gRNA will shed light on these findings.

In conclusion, in this study we provide a fuller and more precise definition of cassette-associated gRNA genes in *T. brucei* that is consistent with analyses of its gRNA transcriptome (Koslowsky et al. 2014; Kirby et al. 2016). Our in-depth analysis of the structural and sequence differences between canonical and noncanonical, and expressed and nonexpressed gRNA genes suggests a possible origin for noncanonical genes from formerly canonical genes. Such knowledge will be invaluable in understanding the evolutionary processes that shape the complexity of *T. brucei*’s kinetoplast genome.

## MATERIALS AND METHODS

### Cell lines and kDNA and small RNA sequencing and processing

Production of the DNA and RNA sequencing reads used in this study, including detailed methods of culturing of trypanosomes, purification, and sequencing of kDNA, assembly of the maxicircle, and purification and sequencing of small mitochondrial RNA transcripts, has been described in Cooper et al. (2019); the present study is based on the same biomaterials. The key procedures are briefly outlined here. Modifications and refinements to the analysis in Cooper et al. (2019) and new analyses are detailed in the following sections.

Kinetoplast DNA was purified from *T. brucei* EATRO 1125 AnTat 1.1 90:13 (wild-type or L262P ATPase subunit γ replacement; Engstler and Boshart 2004; Dewar et al. 2018) as described in Perez-Morga and Englund (1993) with modifications based on helpful advice from Michele Klingbeil. DNA was fragmented to generate ∼550 bp inserts and sequenced to generate paired-end Ilumina MiSeq 300 bp reads (Edinburgh Genomics). Sequence data are available at https://doi.org/10.6084/m9.figshare.7756808.v1. Reads were quality checked and trimmed using fastqc (http://www.bioinformatics.babraham.ac.uk/projects/fastqc/) and Trimmomatic (Bolger et al. 2014) (bases below Q15 were removed). Reads corresponding to the nuclear megabase-sized chromosomes were removed by alignment to the published *T. brucei* TREU 927 genome (Berriman et al. 2005; v5.2; obtained from http://www.genedb.org/) with bowtie2 (Langmead and Salzberg 2012), using default parameters.

Contigs were originally assembled using Velvet (Zerbino and Birney 2008) and a 16.4 kb fragment that includes all known maxicircle genes was identified by similarity searching using ublast (Edgar 2010) against the published, 23.0 kb maxicircle sequence from *T. brucei* strain Lister 427 (M94286.1). The 16.4 kb EATRO 1125 maxicircle fragment was submitted to GenBank (accession number MK584625).

Small RNAs were prepared from crude mitochondrial fractions from ∼10^9^ long slender BSF cells of *T. brucei* EATRO 1125 AnTat1.1 90.13 (WT ATPase subunit γ replacement), and from the same number of PCF cells that had been obtained after differentiation of BSF cells to stumpy forms in mice, parasite purification from blood, and induction of differentiation to PCF in vitro with 6 mM *cis*-aconitate (Dewar et al. 2018). Total cellular RNA was isolated with TRIzol and small RNAs were prepared using the PureLink miRNA Isolation kit (Thermo Fisher). Illumina small RNA libraries were generated following the manufacturer's instructions and sequenced using an Illumina HiSeq platform to generate 125 bp paired reads (Centre for Genomics Research, University of Liverpool). Barcode separated libraries were quality checked using fastqc and adapter-trimmed with cutadapt version 1.9.1 (Martin 2011). Reads that mapped to the nuclear genome were discarded. The remaining reads were mapped to both sense and antisense strands of assembled minicircles using CLC Genomics Workbench v4.9, and alignments were saved as bam files.

### Minicircle assembly

For this study, minicircles were assembled de novo using the Megahit assembler (https://github.com/voutcn/megahit) rather than Velvet (Zerbino and Birney 2008) used in Cooper et al. (2019). The KOMICS package (https://github.com/FreBio/komics; Van den Broeck et al. 2020) was used to automate the assembly of minicircles with contigs containing the conserved minicircle 12-mer CSB-3 (GGGGTTGGTGT or alternative version GGGGTTGATGT that was previously found in a few minicircles [Cooper et al. 2019]). Two independent assemblies were conducted with k-list set to 119 or 255, respectively (kmin and kmax options in KOMICS). All minicircles were oriented in the same orientation, with the 5′ end of conserved minicircle CSB-1 site defined as position 1. Minicircles more than 95% identical (excluding the conserved sequence region) were merged using vsearch cluster_fast with parameters id = 0.95 and qmask = none.

To determine the quality of assemblies, input reads were mapped onto the assembled minicircles using the BWA program “bwamem” with -k 30 and other parameters set to default (Li 2013). Each minicircle was checked manually using IGV; minicircles with any site with fewer than five mapped reads, or with instances where forward and reverse reads map to different minicircles, which may indicate misassemblies, were discarded. All assembled minicircles are available in a Genbank file on Figshare (https://doi.org/10.6084/m9.figshare.16885363).

### Canonical and noncanonical gRNA gene identification

Published edited mRNA sequences were corrected with reference to the assembled EATRO 1125 maxicircle sequence (MK584625). Illumina reads available from an EATRO 1125 whole-cell RNA sequencing project (Silvester et al. 2018) were aligned to these edited sequences using CLC Genomics Workbench v4.9 and the alignments inspected for indications of potential differences from the published editing patterns (Cooper et al. 2019). This analysis suggested two alternative versions for A6 and ND8 mRNAs (Cooper et al. 2019). Guide RNA genes were identified for the twelve edited mRNAs and the alternative versions of A6 and ND8.

Identification of canonical gRNA genes was similar to that reported in Cooper et al. (2019), with some refinements. Code is available at https://github.com/nicksavill/kDNA-annotation. Sense (coding) and antisense (template) strands of assembled maxicircles and minicircles were aligned (not permitting gaps) to edited mRNA to predict canonical gRNAs inside and outside of cassettes. Each circle's coding and template strands were split into 120 nt fragments with each fragment overlapping by 60 nt. Given that gRNA genes are typically 40–50 nt long and almost never exceed 60 nt (Koslowsky et al. 2014), this overlap was deemed sufficient to capture all gRNAs. Each fragment was then aligned to each edited mRNA sequence. The following criteria were used to identify putative canonical gRNA genes: alignments must (i) include at least one U insertion or deletion, (ii) contain a 5′ anchor of at least six consecutive Watson–Crick base pairs, (iii) contain a minimum of 24 matches (Watson–Crick or GU base pairs) with no contiguous mismatches, and (iv) contain no more than three mismatches. As well as identifying probable gRNA genes, these four criteria also identified several thousand low-quality alignments likely to be false positives (e.g., short length with 3 mismatches or short length with short anchor). To filter out these highly likely false positives, any alignments meeting any two of the following three criteria were discarded: (i) less than 27 nt long, (ii) anchor length less than 8 nt, and (iii) containing three mismatches.

In Cooper et al. (2019), noncanonical gRNA genes were identified, and their positions determined, using a nucleotide bias scoring scheme. This method demonstrated that all cassettes encoded either a canonical or noncanonical gene (Cooper et al. 2019). We do not use this method here. Instead, the positions of expressed canonical and noncanonical genes are determined by the mapping of small RNA transcripts as discussed below. The positions of nonexpressed canonical genes are defined by the anchor and guiding regions. The positions of nonexpressed noncanonical genes are not determined; however, their nucleotide frequency structure is very similar to the other genes and thus their positions are also similar (lower panels in Fig. 6). All alignments are available as plain text files on Figshare (https://doi.org/10.6084/m9.figshare.16885363) and as a JBrowse instance on http://hank.bio.ed.ac.uk.

### Guide RNA expression analysis

#### Predicting gRNA gene expression

**FIGURE 13.**
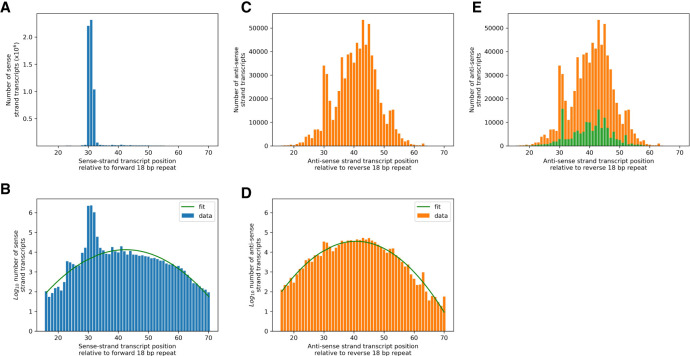
(*A*) Distribution of positions of mapped 5′ templated ends of all cassette-associated sense strand transcripts relative to the upstream 3′ end of the forward 18 bp repeat. Ninety-four percent of transcripts map to positions 30–32 nt. (*B*) As 11A but plotting log_10_ frequency on *y*-axis to reveal the low-level background distribution of transcript 5′ ends. (*C*,*D*), same as for *A* and *B* but for antisense strand transcripts relative to the downstream 5′ end of the reverse 18 bp repeat. Eleven percent of antisense transcripts map to positions 30–32 nt. These are mostly associated with the few antisense encoded canonical gRNA genes (*E*, orange bars: antisense transcripts of antisense canonical gRNA genes, green bars: all other antisense transcripts). The background distribution of sense strand transcripts is approximately normally distributed with mean 42.3 nt and standard deviation 8.5 nt (green line; the normal curve is transformed to a quadratic under log-frequency). Similarly, the background distribution of antisense strand transcripts is approximately normally distributed with mean 40.6 nt and standard deviation 7.3 nt (green line). The non-normal tails of the distributions have been trimmed.

To validate gRNA genes, mitochondrial small RNA transcripts from BSF and PCF cells were pooled and mapped to minicircles using CLC Genomics Workbench v4.9. Previously (Cooper et al. 2019), expression status of gRNA genes (i.e., expressed or nonexpressed) was determined by plotting depth values from the alignment bam files and applying a subjectively assigned cutoff read depth (0.025%), adjusted for the relative frequency of the minicircle in the sample of interest (Cooper et al. 2019). A new, statistical approach was developed based on the observations that (i) the 5′ templated ends of 94% of transcripts mapping to the sense strand within cassettes map 30–32 nt downstream from the 3′ end of the forward 18 bp repeat (Fig. 13A,B; Pollard et al. 1990; Cooper et al. 2019); and that (ii) a similar (but smaller) peak of 5′ templated ends of transcripts mapping to the antisense strand within cassettes exists and is 30–32 nt upstream of the reverse 18 bp repeat (Fig. 13C,D; Cooper et al. 2019). This peak is associated with the few canonical gRNAs encoded on the antisense strand (Fig. 13E). We define the regions 30–32 nt downstream from forward repeat and upstream of the reverse repeat *transcription initiation regions*.

These two peaks in sense and antisense transcripts reside within background distributions that are revealed when transcript abundance is plotted on a log_10_ scale (Fig. 13B,D). These background distributions are remarkably similar: Both have approximately normal distributions (green lines); the background distribution of sense transcripts has a mean of 42.3 nt and a standard deviation of 8.5 nt, and the background distribution of antisense transcripts has a mean of 40.6 nt and a standard deviation of 7.3 nt. There are about 320,000 transcripts in the sense strand background distribution and 700,000 transcripts in the antisense strand background distribution. The origin of these background distributions is unclear, partly because of the method used to process and sequence small RNAs (Cooper et al. 2019) which did not distinguish between primary transcripts and products of nucleolytic cleavage. Whatever their origin, these distributions could be considered noise, and the peaks within the transcription initiation regions as signals of expressed gRNA genes. The problem of classifying a gRNA gene's expression status then becomes one of assessing the size of its transcript signal in relation to the background noise.

We propose a new method to classify each gRNA gene's expression status based on the size of its signal within its cassette with respect to a background level of noise characterized by the empirical background distributions in Figure 11B and D. A caveat of this approach is that the underlying assumption that functionality of a gRNA requires steady-state levels above a background could be wrong, as it has been suggested that gRNAs might get consumed in the editing process (Read et al. 2016); hence, low abundance of a gRNA could conceivably be a consequence of rapid use and turnover during editing. Another caveat, as mentioned above, is that our processing of the small RNAs (see Cooper et al. 2019) did not distinguish between transcripts with 5′ triphosphates and those with 5′ monophosphates, that is, it does not allow us to state definitively that the 5′ ends of mapped gRNAs are transcription start sites. Whether our proposed method is valid or not can be judged by whether expression status correlates with other characteristics of gRNA genes (such as whether they are canonical or not or by their initiation sequences) and the structure of the cassettes they are encoded in (such as their size).

We used the following mathematical approach to determine expression status. A gRNA gene (canonical or noncanonical) is designated as “expressed” if more transcripts than expected by random chance have 5′ ends mapping within the transcription initiation region. Otherwise, it is designated as “nonexpressed.” Let *p* be the probability of a transcript's 5′ end mapping within the transcription initiation region under the null hypothesis of no gene expression in a cassette. We assume that the empirical background distributions of mapped 5′ ends of transcripts (Fig. 13B,D) are the null distributions under the null hypothesis, that is, transcripts of a nonexpressed gRNA gene are randomly distributed according to these background distributions. The probabilities *p*_sense_ and *p*_anti-sense_ are obtained from the empirical background distributions in Figure 13B and D; they are given by the area under the normal curve between 30 and 33, i.e., *p* = Φ((33 − *μ*)/*σ*) − Φ((30 − *μ*)/*σ*) where Φ is the normal cumulative distribution function. The probability *p*_sense_ is estimated to be 0.062 and *p*_anti-sense_ is estimated to be 0.075.

For each cassette, (i) let *N* be the total number of transcripts whose 5′ ends map between the cassette's 18 bp inverted repeats, and (ii) let *R* be the number of those transcripts whose 5′ ends map within the cassette's transcription initiation region. Let *P* be the probability of observing at least *R* transcripts given *N* transcripts. Under the null hypothesis of no gene expression, the number of transcripts, *X*, whose 5′ ends map within the transcription initiation region is binomially distributed with parameters *N* and *p*. Therefore, *P* = Pr(*X* ≥ *R*) = 1 − Pr(*X* < *R*) = 1 − Pr(*X* ≤ *R* − 1) = 1 − *F*_*X*_(*R* − 1;*N*, *p*) where *F*_*X*_ is the binomial cumulative distribution function. A small value of *P* indicates a greater than expected number of transcripts whose 5′ ends map within the cassette's transcription initiation region under the null hypothesis of no gene expression. A gRNA gene's expression status if determined using a significance level of α = 0.05 corrected using the Holm method for multiple tests.

Conserved transcription initiation regions for the eleven orphan gRNA genes have not been found. This means that the above analysis cannot be applied to them. Transcripts do map to all of the orphan genes; ranging from three to several thousand suggesting some, at least, are likely to be expressed, especially since several of these genes encode nonredundant gRNAs. Without any statistical method to determine expression, their expression status is based subjectively on the number of transcripts that map to their 5′ ends. Their relatively small number compared to cassette-encoded gRNA genes means they have negligible effect on the main conclusions of this study.

#### Predicting the positions of expressed gRNA genes

In Cooper et al. (2019), the positions of expressed gRNA genes on minicircles were determined by visual inspection of the alignment bam files. Here, a statistical approach is now used to determine gene position. The 5′ start position of an expressed gRNA gene (canonical or noncanonical) is defined as the position with the greatest number of mapped transcript 5′ ends within the transcription initiation region. The 3′ end position of an expressed gRNA gene is defined as the 90th-percentile position of the mapped 3′ ends of only those transcripts with an untemplated oligo(U)-tail. This definition is used because it results in the smallest standard deviation in the distance between the end position of an expressed canonical gRNA gene and the end position of its cognate canonical gRNA gene found by alignment to edited mRNA (see Supplemental Fig. S1 for a discussion and comparison of 3′ end position estimators).

All cassettes, canonical gRNA genes (expressed and nonexpressed) and expressed gRNA genes (canonical and noncanonical) can be found in Supplemental Files S1, S2, and S4, respectively.

## DATA DEPOSITION

Sequence data are available at https://doi.org/10.6084/m9.figshare.7756808.v1 from the pleomorphic, laboratory-adapted cell line *T. brucei* EATRO 1125 AnTat1.1 90:13. All alignments are available as plain text files on Figshare (https://doi.org/10.6084/m9.figshare.16885363) and as a JBrowse instance on http://hank.bio.ed.ac.uk. Scripts for bioinformatics analyses are available in the GitHub repository https://github.com/nicksavill/kDNA-annotation.

## SUPPLEMENTAL MATERIAL

Supplemental material is available for this article.

## Supplementary Material

Supplemental Material
