# Supplementary material for: Organization of minicircle cassettes and guide RNA genes in Trypanosoma brucei
Source: RNA. 2022 Jul;28(7):972–92. doi: 10.1261/rna.079022.121 (PMC9202587; doi:10.1261/rna.079022.121)
Supplement: Supplemental Material [file supp_28_7_972__DC1.html]

Organization of minicircle cassettes and guide RNA genes in Trypanosoma brucei — Supplemental Material 

# Organization of minicircle cassettes and guide RNA genes in *Trypanosoma brucei*

## Supplemental Material

- Supplemental\_Fig\_S1.jpeg
- Supplemental\_Fig\_S2.jpeg
- Supplemental\_File\_S1.csv
- Supplemental\_File\_S2.csv
- Supplemental\_File\_S3.csv
- Supplemental\_File\_S4.csv
