# Supplementary figures and images for: Organization of minicircle cassettes and guide RNA genes in Trypanosoma brucei
Source: RNA. 2022 Jul;28(7):972–92. doi: 10.1261/rna.079022.121 (PMC9202587; doi:10.1261/rna.079022.121)

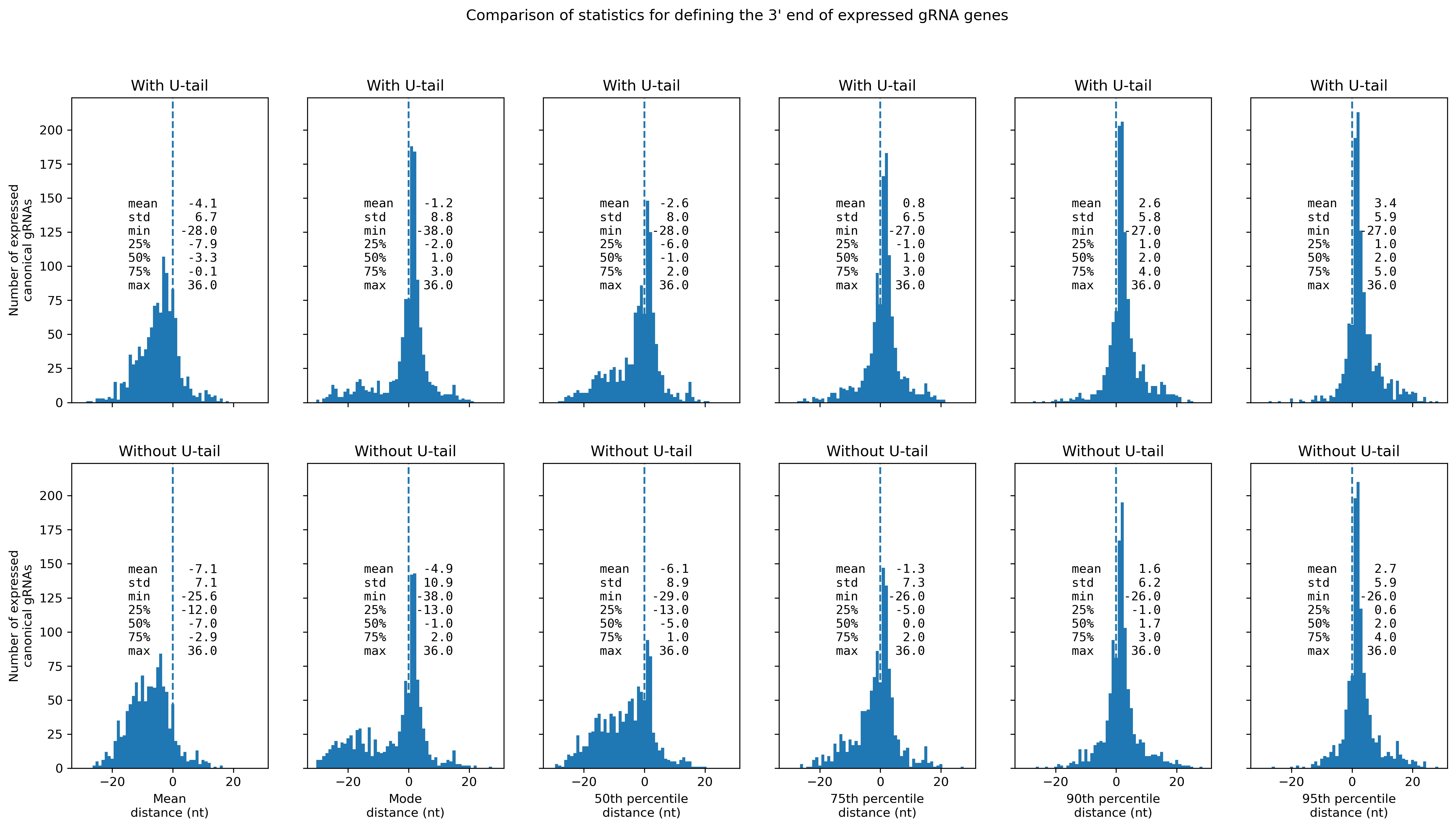

Supplement: Supplemental Material [file supp_079022.121_Supplemental_Fig_S1.jpg]

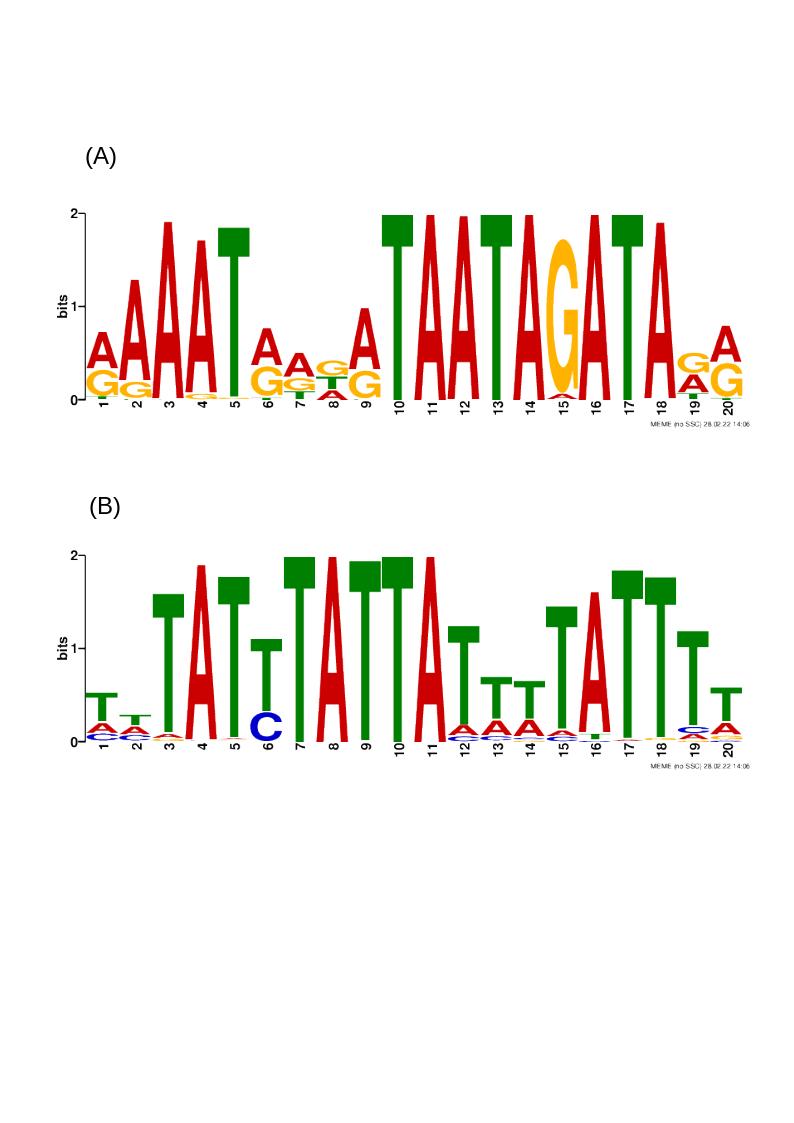

Supplement: Supplemental Material [file supp_079022.121_Supplemental_Fig_S2.jpg]
